# Serum inflammatory proteomic signatures define chronic inflammatory demyelinating polyneuropathy and inform on disease activity

**DOI:** 10.1016/j.ebiom.2026.106348

**Published:** 2026-06-25

**Authors:** Amol Keshavasa Bhandage, Frauke Stascheit, Hannah Preßler, Sarah Hoffmann, Andreas Meisel, Anna Rostedt Punga

**Affiliations:** a Department of Medical Sciences, Clinical Neurophysiology, Uppsala University, Uppsala, Sweden; b Department of Neurology with Experimental Neurology, Charité - Universitätsmedizin Berlin, Corporate Member of Freie Universität Berlin and Humboldt Universität zu Berlin, Berlin, Germany; c Neuroscience Clinical Research Center, Charité - Universitätsmedizin Berlin, Freie Universität Berlin and Humboldt Universität zu Berlin, Berlin, Germany; d Center for Stroke Research Berlin, Charité — Universitätsmedizin Berlin, Corporate Member of Freie Universität Berlin, Humboldt-Universität zu Berlin, Germany; e Clinical Neurophysiology, Uppsala University Hospital, Uppsala, Sweden

**Keywords:** Chronic inflammatory demyelinating polyneuropathy, CIDP, Proteomic biomarkers, Neuroimmune, IVIG treatment

## Abstract

**Background:**

Chronic Inflammatory Demyelinating Polyneuropathy (CIDP) is an immune-mediated neuropathy with heterogeneous presentation and variable treatment response. Blood-based biomarkers remain lacking, and interpretation is confounded by widespread immunoglobulin (IG) therapy. The primary aim was to identify serum inflammatory proteomic signatures associated with CIDP; exploratory aims included associations with disease activity, phenotype, and treatment response.

**Methods:**

In this case–control study, 92 proteins were quantified using multiplex proximity extension assay and ELISA in 358 sera samples from patients with CIDP, multiple sclerosis (MS), and IG-treated myasthenia gravis (MG) as well as age- and sex- matched healthy controls (HC). Logistic regression with Bonferroni correction, principal component analysis, and correlation analyses were applied to identify differential expression across diagnoses and CIDP subgroups.

**Findings:**

Seven proteins, TNFSF14, CD40, SIRT2, CCL3, IL8, TGFα, and uPA, consistently distinguished CIDP from HC, MS, and IG-treated MG, representing disease-specific signatures independent of IG exposure. Six additional proteins (STAMBP, CASP-8, EN-RAGE, OSM, HGF, and CXCL6) were IG-responsive. CXCL9, CDCP1, and CCL20 correlated with muscle weakness, while calprotectin and neurofilament light chain reflected broader inflammatory and axonal injury signatures. Patients with unstable active disease exhibited higher CCL4, IL-8, CCL3, FGF-21, and IL-17A. IL-8, EN-RAGE, and CASP-8 predicted clinical improvement, and IL-5 tracked longitudinal disability changes.

**Interpretation:**

CIDP is characterised by distinct serum inflammatory proteomic signatures comprising disease-specific and IG-responsive signatures. These candidate biomarkers may support improved patient stratification, disease monitoring, and prediction of treatment response, advancing individualised therapy in CIDP.

**Funding:**

Erling-Persson Foundation, Sweden (#2022_0030 to ARP) and the German Research Foundation, Germany (KFO5023 TP8 to AM).

**Research in context**

**Evidence before this study**

Chronic Inflammatory Demyelinating Polyneuropathy (CIDP) lacks reliable blood-based biomarkers that reflect disease activity, capture clinical heterogeneity, or predict treatment response. Previous studies have identified candidate markers—such as serum neurofilament light chain, calprotectin, and selected cytokines—but with limited disease specificity, and are often confounded by widespread immunoglobulin (IG) therapy. Furthermore, prior work has rarely incorporated treatment-matched disease controls, making it difficult to distinguish CIDP-intrinsic inflammation from IG-driven changes.

**Added value of this study**

This study provides the most comprehensive characterisation of serum inflammatory signatures in CIDP to date. We identify a robust 13-protein signature with high diagnostic accuracy and refine it into seven CIDP-specific biomarkers (TNFSF14, CD40, SIRT2, CCL3, IL8, TGFα, and uPA). In parallel, we define six IG-modulable proteins (STAMBP, CASP-8, EN-RAGE, OSM, HGF, and CXCL6), thereby enabling the separation of therapy-related from disease–intrinsic pathways. We also highlight IL-8, EN-RAGE, and CASP-8 as the first serum-based potential predictors of subsequent clinical improvement in CIDP. Inflammatory markers further correlated with muscle weakness, disability, age-related immune variation, and axonal injury, offering new mechanistic insight into clinical heterogeneity.

**Implications of all the available evidence**

These findings demonstrate that CIDP is characterised by distinct, biologically coherent inflammatory serum signatures. By differentiating IG-independent from IG-responsive pathways, this study provides a framework for more accurate biomarker interpretation in both research and clinical practice. The identified markers have the potential to improve patient stratification, disease activity monitoring, and early assessment of treatment responsiveness, supporting the development of personalised therapeutic strategies in CIDP.

## Introduction

Chronic Inflammatory Demyelinating Polyneuropathy (CIDP) is an acquired immune-mediated neuropathy characterised by progressive or relapsing motor and sensory dysfunction, in which both humoural and cell-mediated mechanisms contribute to peripheral nerve demyelination.^1^^,^^2^ A hallmark pathological feature of typical CIDP is macrophage-mediated phagocytosis of myelin, resulting in segmental demyelination.^3^ In contrast, recent studies have identified a distinct subgroup of patients with IgG4 antibodies against paranodal proteins, who exhibit fundamentally different pathogenic mechanisms and clinical characteristics.^4^ Although established clinical and electrophysiological criteria guide diagnosis,^5^ the marked heterogeneity of CIDP and its overlap with other immune-mediated neuropathies continue to complicate diagnosis, prognostication, and longitudinal disease monitoring.^6^, ^7^, ^8^ Current standard-of-care therapies, including corticosteroids, intravenous immunoglobulin (IVIG), and plasma exchange, are effective in many patients; however, a considerable proportion require long-term treatment or experience relapses,^9^ and sustained remission is achieved only in a minority.^10^ With the emergence of targeted immunotherapies, such as FcRn inhibition (e.g., efgartigimod)^11^ and complement inhibition (e.g., riliprubart^12^ and empasiprubart^13^), the need for reliable biomarkers to enable patient stratification and therapeutic monitoring has become increasingly urgent.

Several blood-based biomarkers have been explored in CIDP. Serum neurofilament light chain (sNfL) correlates with axonal damage and shows utility in monitoring treatment response, though diagnostic specificity is limited during ongoing therapy.^14^, ^15^, ^16^ Serum calprotectin (sCLP) and complement factors are elevated and associated with active disease course,^16^^,^^17^ while elevated endothelin-1 correlates with disability and disease severity.^18^ More recently, peripherin^19^ and periaxin^20^ have emerged as potential markers of axonal and Schwann cell and myelin-related pathology, respectively. However, most studies have been limited to small exploratory cohorts without systematic validation across clinical phenotypes, disease stability states, or treatment exposure. Importantly, interpretation of circulating biomarkers is further complicated by immunoglobulin (IG) therapy, which may modulate inflammatory signatures independently of intrinsic disease activity.

Recent advances in proteomic and metabolomic approaches have expanded the CIDP biomarker landscape. Altered bile acid and arachidonic acid metabolite profiles suggest systemic inflammation and microbiota-related alterations.^21^ Multiplex proteomic platforms have identified candidate inflammatory proteins linked to CIDP activity, including IRAK4, SUGT1, DCTN1, NT5C3A, and GLRX^22^ in serum and MMP3, THBD, and ITGAM in plasma.^23^ Distinct serum inflammatory protein profiles separating CIDP from the autoimmune neuromuscular disorder myasthenia gravis (MG) have also been reported.^24^

While cerebrospinal fluid (CSF) and skin biopsy markers, such as sphingomyelin, IL-8, and intraepidermal nerve fiber density, provide mechanistic insight,^25^, ^26^, ^27^ serum biomarkers are particularly attractive for longitudinal monitoring due to their accessibility and scalability. Yet no single biomarker has achieved broad clinical utility, and isolated markers often lack sufficient specificity and are unlikely to capture the immunological complexity of CIDP.^7^^,^^8^^,^^27^

In this study, we aimed to define serum inflammatory proteomic signatures in CIDP using a multiplex proteomics approach. Additionally, we sought to identify proteins associated with clinical phenotype, disease activity and stability, neuroaxonal injury, and treatment exposure. By integrating proteomic profiles with established clinical and biochemical markers, we aimed to identify candidate biomarker signatures with potential utility for patient stratification and therapeutic monitoring in CIDP.

## Methods

### Standard protocol approvals, registrations, and patient consents

The study was approved by the Swedish Ethical Review Authority (permit number 2024-02500-01) and the Ethics Committee of Charité–Universitätsmedizin Berlin (approval number EA4/166/23). Written informed consent was obtained from all participants before inclusion. The study was registered in the German Clinical Trials Register (DRKS00032455).

### Study design and patient cohorts

The primary hypothesis of the study was that CIDP is associated with a distinct inflammatory serum protein signature that differentiates patients from both healthy individuals and neuroimmune disease controls, and that this signature may serve as a foundation for biomarkers related to treatment response and clinical course. The secondary hypothesis was that additional protein signatures independent of IVIG exposure could be identified. All other analyses, including associations with clinical subgroups, electrophysiological subtypes, age, disease activity, and treatment history, were prespecified as exploratory and interpreted as hypothesis-generating. The study funders had no role in the study design, data collection, analysis, interpretation, reporting, or writing of the manuscript.

This case–control cross-sectional study included serum samples from patients with CIDP consecutively recruited at Charité–Universitätsmedizin Berlin between January 2023 and September 2024. CIDP diagnoses were established according to the current European Academy of Neurology/Peripheral Nerve Society (EAN/PNS) criteria,^5^ and only patients with typical CIDP were enrolled. All patients were diagnosed and evaluated by experienced neuromuscular specialists. As part of the diagnostic work-up, patients were screened for relevant differential diagnoses, including monoclonal gammopathies and antibodies against paranodal proteins (e.g., neurofascin-155, contactin-1, and CASPR1), none of which were detected in this cohort.

For comparison, healthy control (HC) samples were obtained from a blood donor biobank at the Transfusion Unit, Uppsala University Hospital. In addition, disease controls from individuals with other neuroimmunological disorders, including multiple sclerosis (MS) and myasthenia gravis (MG) were included. Samples from treatment-naive patients with MS were collected at the time of diagnosis at Uppsala University Hospital. To specifically assess potential IG-related effects on serum inflammatory markers, patients with generalised MG receiving intravenous immunoglobulin (IVIG) therapy were recruited from the Neurology Clinic at Uppsala University Hospital.

Demographic and clinical variables collected for patients with CIDP included sex, age, disease duration, electrophysiological findings, clinical assessments, and ongoing treatment regimens. Sex was extracted from the electronic medical records, where it is routinely documented at the time of clinical registration. Both women and men were included in the study, and sex was incorporated as a covariate in the statistical analyses to account for potential sex-related differences. CIDP subtypes were defined based on nerve conduction studies in accordance with the EAN/PNS diagnostic criteria.^5^ Patients were classified as having *pure demyelinating* CIDP when electrophysiological features fulfilled demyelinating criteria without evidence of significant axonal damage. *Axonal demyelinating* CIDP was defined by the presence of demyelinating features consistent with CIDP together with secondary axonal involvement, such as reduced compound muscle action potential amplitudes, in the absence of criteria for primary axonal neuropathy. Patients who met criteria for primary axonal neuropathy were excluded.

Clinical assessments included the Medical Research Council sum score (MRC-SS) evaluating eight bilateral muscle groups (shoulder abduction, elbow flexion, wrist extension, hip flexion, knee extension, ankle dorsiflexion) with a total score ranging from 0 to 80 (where higher scores indicate greater muscle strength), and the Inflammatory Neuropathy Cause and Treatment Disability score (INCAT-DS).^28^ Disease activity was evaluated using the CIDP Disease Activity Scale (CDAS),^10^ which integrates clinical course, treatment dependency, and recent disease dynamics. CDAS distinguishes ongoing immunologically active disease from residual neurological impairment by emphasising recent progression, fluctuation, or treatment responsiveness rather than fixed deficits. Patients categorised as *active stable* (CDAS 3) or *unstable active* disease (CDAS 5) exhibited evidence of ongoing immune-mediated disease activity, whereas patients in *remission* (CDAS 2) had stable neurological deficits without signs of active disease. Available blood biomarkers included sCLP and sNfL. CSF samples were obtained as part of the routine diagnostic work-up at the time of CIDP diagnosis and were extracted from clinical records for the present analysis.

### Serum sampling and handling

Venous blood samples from all participants were collected using identical standardised pre-analytical procedures. Samples were allowed to clot for 30–60 min at room temperature, centrifuged, aliquoted, and immediately frozen at −80 °C. All serum aliquots were stored at −80 °C under uniform conditions, and no samples underwent repeated freeze–thaw cycles prior to biomarker or proteomic measurements. Centralised batch analyses were applied across all cohorts to minimise potential storage- or batch-related effects on the analytes.

### Serum calprotectin (sCLP) and serum neurofilament light (sNfL) measurements

Serum Calprotectin (sCLP) concentrations were measured using the fCAL turbo test (Cat. No. K190784, Bühlmann Laboratories AG, Schönenbuch, Switzerland) on a COBAS 8000 semi-automated analyser (RRID: SCR_026640, Roche, Germany) according to the manufacturer’s protocol.^29^ Serum Neurofilament Light (sNfL) levels were quantified using the SIMOA Nf-light® assay (Cat. No. 104364, Quanterix Corp., Boston, MA, USA) on the SR-S immunoassay analyser, SIMOA™ (Quanterix Corp., Boston, MA, USA).^30^ Total CSF protein was measured in mg/L.

### Proteomic profiling using proximity extension assay (PEA)

Proteomic profiling was performed using the Olink Target 96 Inflammation panel (Olink Proteomics AB, Uppsala, Sweden, RRID: SCR_003899), which quantifies 92 human inflammation-related proteins. Samples were randomised in a double-blinded manner and distributed evenly across six 96-well plates. Analyses were conducted at the Clinical Biomarkers Facility, Science for Life Laboratory, Uppsala University (RRID: SCR_014078).

The proximity extension assay (PEA) was carried out according to the manufacturer’s instructions. Briefly, serum samples and negative controls were incubated with probe mixtures containing 92 validated antibody pairs, each conjugated to a unique single-stranded DNA oligonucleotide. Dual antibody binding enabled sequence-specific hybridisation and DNA polymerase–mediated extension, generating unique amplicons that were subsequently quantified by high-precision and high-throughput microfluidic real-time PCR.

Cycle threshold (Ct) values were normalised to internal spike-in controls to yield Normalised Protein Expression (NPX) values on a log_2_ scale. Internal controls also served as quality metrics. NPX values were successfully generated for all 92 proteins across all samples.^31^^,^^32^ NPX is a relative, unitless measure reflecting protein abundance rather than absolute concentration. Although the lower limit of detection (LOD) was defined as three standard deviations above background, NPX values below the LOD were retained in the dataset, as low-abundance proteins may still hold biologically relevance.

### Technical validation of shared proteins by Olink Reveal Panel in CIDP and HC samples

The Olink Target 96 inflammation panel and the Olink Reveal Panel (a total of 1034 proteins, including about 600 inflammation-related proteins; Olink Proteomics AB, RRID: SCR_003899) share 68 inflammatory proteins. In serum samples from patients with CIDP and HC, these overlapping proteins were methodologically validated by the Olink Reveal Panel. Similar to the inflammation panel, PEA was performed; however, the final amplification and detection step was carried out using Illumina-based Next-Generation Sequencing (NovaSeq). The primary output consisted of sequence-based counts, which were normalised and converted into NPX values for further data analysis.

### Statistical analysis

All statistical analyses and visualisation were performed using NPX values in GraphPad Prism version 10.6.1 and R version 4.5.1.

#### Primary analyses

To identify proteins distinguishing CIDP from MS, MG, and HC, logistic regression models were fitted using the “lrm” function in the *rms* R package. Both the unadjusted model (model 0) and the model adjusted for age and sex (model 1) were applied. To account for multiple comparisons across the 92 measured proteins, a Bonferroni-corrected significance threshold of *p* < 0.000543 (0.05/92) was applied. Logistic regression analyses were performed on the full 92-protein dataset across all study groups (CIDP, MS, MG, and HC).

Principal Component Analysis (PCA) was applied to the top 28-protein NPX dataset across all cohorts to reduce dimensionality and provide an unsupervised visualisation of global multivariate patterns.

Boruta feature selection was applied solely for technical validation, including confirmation of protein-level robustness in the Olink Inflammation panel and an independent Olink Reveal panel. The Boruta algorithm (“Boruta” R package) introduces permuted shadow features as an internal negative control and identifies confirmed markers based on importance rankings across 200 iterations.^33^ A protein is classified as “confirmed” if its importance score is significantly higher than the maximum shadow feature across 200 iterations, determined by a two-sided binomial test with *p* < 0.01. This built-in significance criterion served as the cut-off for feature retention.

#### Secondary analyses

For analyses of IVIG treatment effects, a more conservative threshold of *p* < 0.01 was used, given the number of comparisons and the predefined nature of this secondary research question.

#### Exploratory analyses

Subgroup comparisons (e.g., sex, electrophysiological subtype, clinical stability, treatment status) and protein-to-age associations were considered exploratory. For these analyses, a threshold of *p* < 0.05 was used.

Associations between continuous variables, including age, were assessed using Spearmanś Rank correlation, with correlation strength categorised as strong (0.70–1.00), moderate (0.40–0.69), or weak (0.10–0.39).^34^ Receiver operating characteristic (ROC) curves were constructed to descriptively evaluate the sensitivity and specificity, as well as the predictive performance, of individual proteins or combined signatures.

### Role of funders

The funders of the study had no role in study design, data collection, data analysis, data interpretation, or the writing of the report.

## Results

### Clinical characteristics

This study included serum samples from 51 patients with CIDP, 93 patients with MS, 22 patients with IVIG-treated MG, and 192 HCs. Among CIDP patients, 61% (n = 31) were male with a median age of 64.5 years (IQR 56.0–74.3) at sampling, compared with 48% male among HC (n = 93; median age: 52.0 years; IQR 42.3–61.0). Baseline demographics and clinical characteristics are summarised in Table 1. The median time from symptom onset to treatment initiation was 8 months (IQR 2.0–24.0).

**Table 1:** Clinical and demographical characteristics of patients with CIDP and HC.

	CIDP	HC	MS	MG
Number, N	51	192	93	22
Sex, Male, N (%)	33 (65%)	93 (49%)	34 (36.6%)	6 (27.3%)
Age at Manifestation, Median (IQR)	57.0 (46.5–67.5)	–	41.0 (30.0–52.0)	37.0 (27.5–55.5)
Age at diagnosis, Median (IQR)	58.0 (48.0–70.5)	–	41.0 (30.0–52.0)	37.0 (27.5–55.5)
Age at sampling, Median (IQR)	64.5 (56.8–77.3)	52.0 (42.3–61.0)	41.0 (30.0–52.0)	47.0 (38.0–66.0)
Total INCAT score at sampling, Median (IQR); (missing values)	2.0 (2.0–4.0); 0		–	–
MRC-sum score at sampling, Median (IQR); (missing values)	74.0 (63.3–78.0); 11	–	–	–
CDAS Classification, n (%)				
Remission (CDAS 2)	2 (4%)	–	–	–
Active stable (CDAS 3)	34 (67%)			
Unstable active (CDAS 5)	15 (29%)			
Cerebrospinal fluid				
Cell number, Median (IQR)	2.0 (1.8–3.0)	–	–	–
Protein level (mg/l), Median (IQR)	785.5 (565–1325)			
(missing values)	4			
Neurography, N (%)^a^				
Pure demyelinating	42 (82%)	–	–	–
Axonal-demyelinating	9 (18%)			
(missing values)	0			
Immunotherapy exposure, N (%)				
Therapy naïve	0 (0%)	–	–	–
Intravenous Immunoglubilines	40 (78%)	–	–	22 (100%)
Subcutaneus Immunoglobulines	22 (43%)	–	–	–
Rituximab	9 (18%)	–	–	–
(missing values)	0	–	–	–
Time to first treatment in months, Median (IQR)	8 (2.0–24.0)	–	–	–
Biomarker				
Serum Calprotectin (μg/ml), Median (IQR)	1.5 (0.95–2.6)	–	–	–
sNfL (pg/ml), Median (IQR)	17.5 (9.6–25.0)	–	–	–
(missing values)	13	–	–	–

Data are median (IQR) and n (%) for the baseline variables. *Abbreviations*: CIDP-chronic inflammatory demyelinating polyneuropathy, CDAS = Chronic Inflammatory Demyelinating Polyneuropathy Disease Activity Status, CLP = serum calprotectin, HC = healthy controls, INCAT-inflammatory neuropathy cause and treatment sensory sum score, IQR = interquartile range, MRC = medical research council sum score, sNfL = serum neurofilament light chain, – = not applicable.

a According to EAN/PNS-Guidelines from 2021.^5^

Treatment regimens were consistent with current clinical practice. At the time of sampling, most patients were receiving immunoglobulin (IG) therapy, either intravenously (IVIG, n = 40) or subcutaneously (SCIG; n = 22). SCIG was typically initiated as maintenance therapy following or prior to IVIG exposure; therefore, several patients in the SCIG group had previously received IVIG earlier in their disease course. Consequently, treatment categories reflect therapy exposure over the disease course rather than mutually exclusive groups. No patient received IVIG or SCIG concurrently at the time of sampling. An additional subset of patients (18%, n = 9) was treated with rituximab instead of IG therapy. Prior immunotherapies, including corticosteroids, were common earlier in the disease course, consistent with standard CIDP treatment algorithms. Given the real-world observational design, no predefined washout period was applied, and prior treatment exposures were heterogeneous. Treatment-related subgroup analyses, therefore, focused on immunotherapy status at the time of sampling.

Based on the CDAS, 34 patients (67%) had active stable disease (CDAS 3), 15 patients (29%) had unstable active disease (CDAS 5), and two patients (4%) were in remission (CDAS 2). Disease severity was moderate, with a median INCAT disability score of 2.0 (IQR 2.0–4.0) and a median MRC-sum score of 74 (IQR 63.3–78.0). Follow-up clinical assessments, including INCAT disability scores, were available for a subset of patients and were used to evaluate changes in disability after serum sampling.

### CIDP-specific inflammatory serum protein biomarkers

**Fig. 1:**
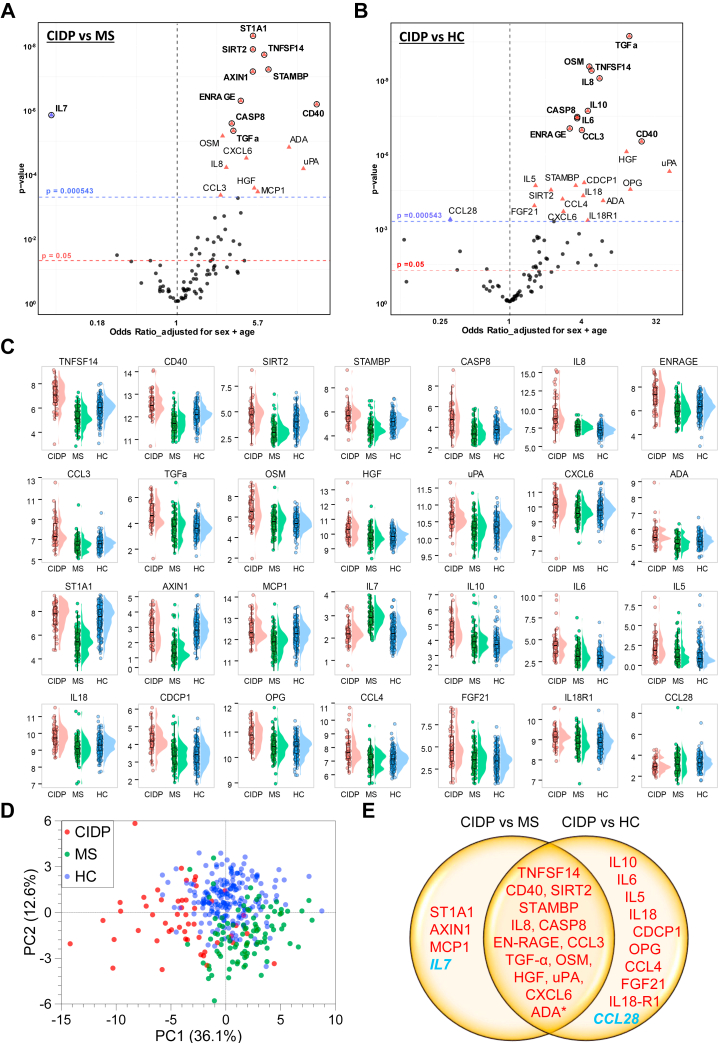
**28 inflammatory proteins differentiate CIDP from MS and/or HC.** Volcano plot for (A) CIDP vs. MS, and (B) CIDP vs. HC comparison, presenting OR with CIDP as outcome, and *p*-values obtained in logistic regression Model 1, adjusted for age and sex. Red and blue lines indicate *p* = 0.05 and *p* = 0.000543 (0.5/92), respectively, and the top 10 proteins in each volcano plot are marked with a black circle. Logistic regression analysis with ORs, 95% CIs, *p*-values, and C-statistics for all proteins is presented in Supplementary Table S1. (C) Raincloud plot and (D) PCA plot of the top 28 proteins significantly different in the CIDP vs. MS/HC groups. (E) Venn diagram representing the top 14 proteins statistically significantly higher in CIDP sera compared to both MS and HC sera; red, higher in CIDP sera; italic blue, lower in CIDP sera; ADA^∗^, ADA could not be methodologically validated.

**Supplementary Fig. S1:**
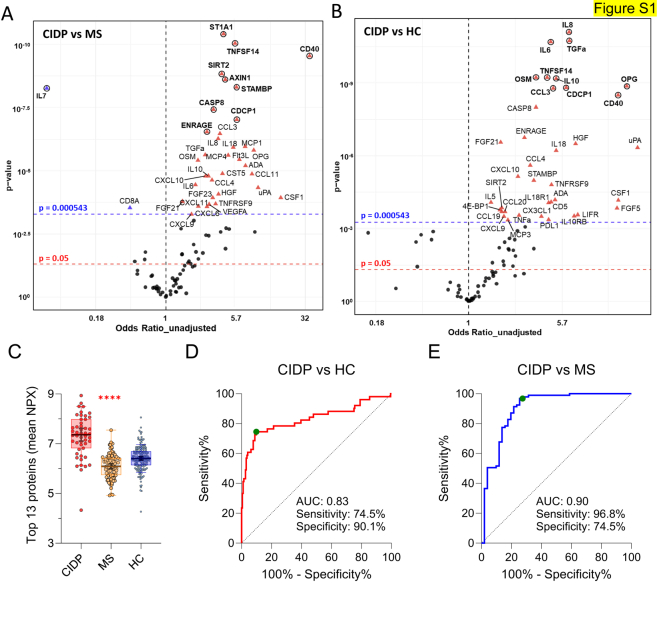
**Fig S1: Sensitivity and specificity of top 25 proteins.** (A) Volcano plot for CIDP vs. MS and (B) CIDP vs. HC comparison presenting OR with CIDP as outcome, and *p*-values obtained in logistic regression Model 0, unadjusted for age and sex. Red and blue lines indicate *p* = 0.05 and *p* = 0.000543 (0.5/92), respectively, and top 10 proteins in each volcano plot are marked with black circle. (C) Box plot with jitter dots presenting mean NPX of top 13 proteins differing in CIDP vs. MS/HC/IVIG-MG comparison. ROC curves presenting high sensitivity and specificity with Youden’s index (green dot) of top 13 proteins in respective comparisons, (D) CIDP vs. HC, and (E) CIDP vs. MS.

To determine a disease-specific serum protein signature, we performed logistic regression, adjusted for age and sex and corrected for multiple testing (Bonferroni threshold *p* < 0.000543), identified top 26 inflammatory proteins significantly elevated in CIDP compared with MS and/or HC, with the exceptions of IL7, which was elevated in MS, and CCL28, which was lower than in HC (Fig. 1A–C; Supplementary Fig. S1; Supplementary Table S1). Proteins significantly elevated in CIDP compared with MS included: ST1A1, SIRT2, TNFSF14, AXIN1, STAMBP, ENRAGE, CD40, CASP8, TGFα, OSM, ADA, CXCL6, IL8, uPA, HGF, MCP-1, and CCL3 (Fig. 1A and C). Compared with HC, CIDP sera showed increased levels of: TGFα, OSM, TNFSF14, IL8, IL10, CASP8, IL6, ENRAGE, CCL3, CD40, HGF, uPA, IL5, STAMBP, CDCP1, OPG, IL18, SIRT2, CCL4, ADA, FGF21, CXCL6, and IL18-R1 (Fig. 1B and C).

PCA with these top 28 proteins reflected how a broader proteomic signature, rather than isolated markers, could represent the separation of individuals among the groups (Fig. 1D). A distinct 14-protein signature elevated in CIDP relative to both MS and HC consisted of: TNFSF14, CD40, SIRT2, STAMBP, CASP8, EN-RAGE, CCL3, IL8, TGF-α, OSM, ADA, HGF, uPA, and CXCL6 (Fig. 1E).

Several markers showed apparent group differences in unadjusted analysis that were attenuated after adjusting for age and sex (Supplementary Fig. S1A and B; Supplementary Table S1).

### Validation using the Olink Reveal Panel

**Supplementary Fig. S2:**
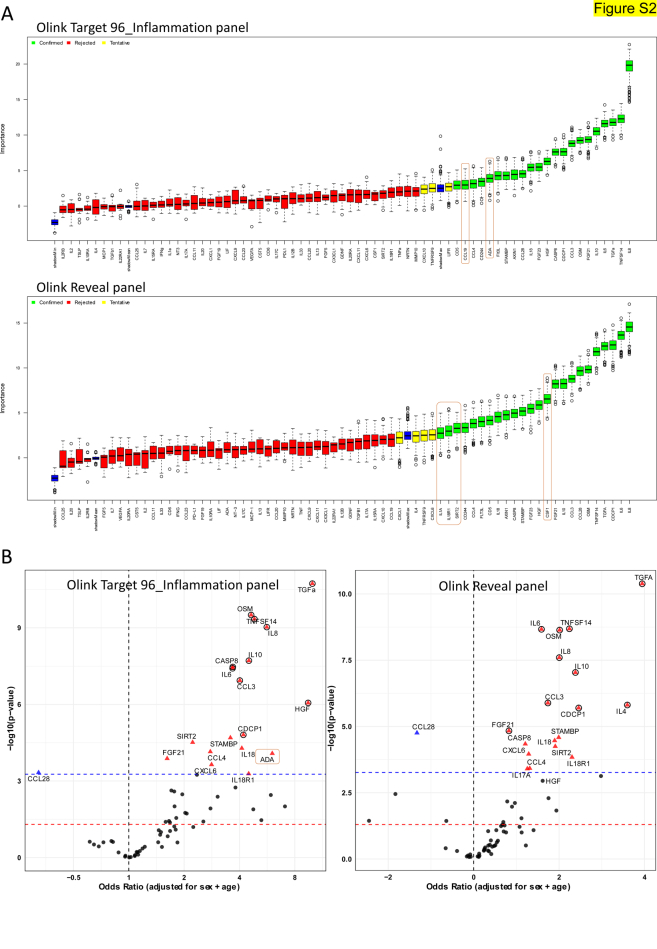
**Fig S2: Methodological validation of 68 shared inflammatory proteins in sera from patients with CIDP and HC.** (A) Boruta algorithm analysis, and (B) volcano plot, for 68 shared inflammatory proteins within the Olink Target 96 Inflammation panel and Olink Reveal panel, presenting OR with CIDP as outcome in CIDP vs. HC comparision, and *p*-values obtained in logistic regression Model 0, unadjusted for age and sex. Blue and red line indicates *p* = 0.05 and *p* = 0.000543 (0.5/92), respectively, and top 10 proteins in each volcano plot are marked with black circle.

To validate the robustness of our findings, CIDP and HC sera were also analysed using the Olink Reveal Panel, which includes 68 proteins overlapping with the T96 inflammation panel. Boruta analysis and age- and sex-adjusted logistic regression on these 68 proteins indicated that ADA did not replicate across panels (Supplementary Fig. S2A and B). ADA was therefore removed from the original 14-protein signature (Fig. 1E), yielding a refined set of 13 CIDP-associated serum markers.

ROC-based classification using these 13 proteins demonstrated a greater separation between CIDP and HC/MS, with a high Youden’s index reaching a sensitivity of 74.5% and a specificity of 90.1% with HC and a sensitivity of 96.8% and a specificity of 74.5% with MS (Supplementary Fig. S1C–E).

### CIDP-specific vs. immunoglobulin therapy-related effects

**Fig. 2:**
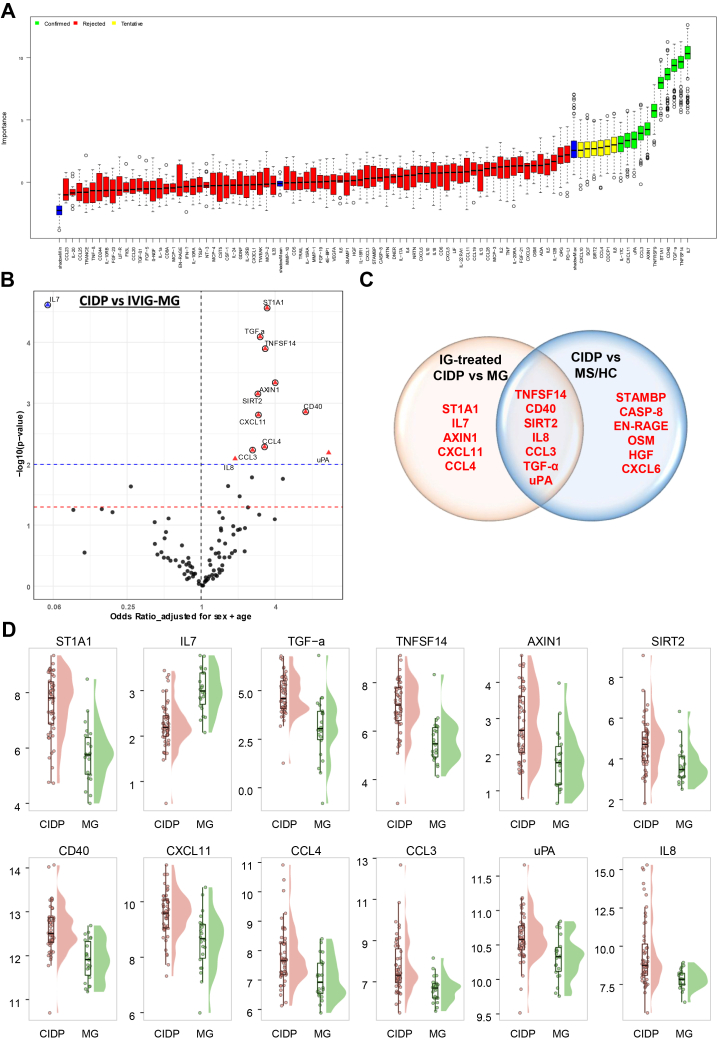
**Seven CIDP-specific proteins unrelated to immunoglobulin.** (A) Boruta plot, and (B) volcano plot for CIDP vs. IVIG-MG comparison, presenting OR with CIDP as outcome, and *p*-values obtained in logistic regression Model 1, adjusted for age and sex. Red and blue lines indicate *p* = 0.05 and *p* = 0.01, respectively, and the top 10 proteins in the volcano plot are marked with a black circle. (C) Venn diagram representing the division of top 13 proteins differentiating CIDP from MS and HC into IVIG-modulable or IVIG-independent markers; red, higher in CIDP sera. (D) Raincloud plot showing individual proteins differing between CIDP and IVIG-treated MG.

To distinguish CIDP-specific effects from potential IG therapy-related effects, we included sera from 22 IVIG-treated patients with MG. Comparative analysis revealed elevated levels of 11 proteins (ST1A1, TGF-α, TNFSF14, AXIN1, SIRT2, CD40, CXCL11, CCL4, CCL3, uPA, and IL8) and lower levels of IL7 in CIDP compared with IVIG-treated MG (Fig. 2A, B, D).

Among the 13 CIDP-elevated serum markers (Fig. 1E), seven consistently distinguished CIDP from all three comparator groups (HC, MS, and IVIG-treated MG): TNFSF14, CD40, SIRT2, CCL3, IL8, TGFα, and uPA. In contrast, STAMBP, CASP-8, EN-RAGE, OSM, HGF, and CXCL6 appeared to be modifiable by IVIG treatment (Fig. 2C).

### Inflammatory serum proteins associate with age, muscle strength, and disease activity

**Fig. 3:**
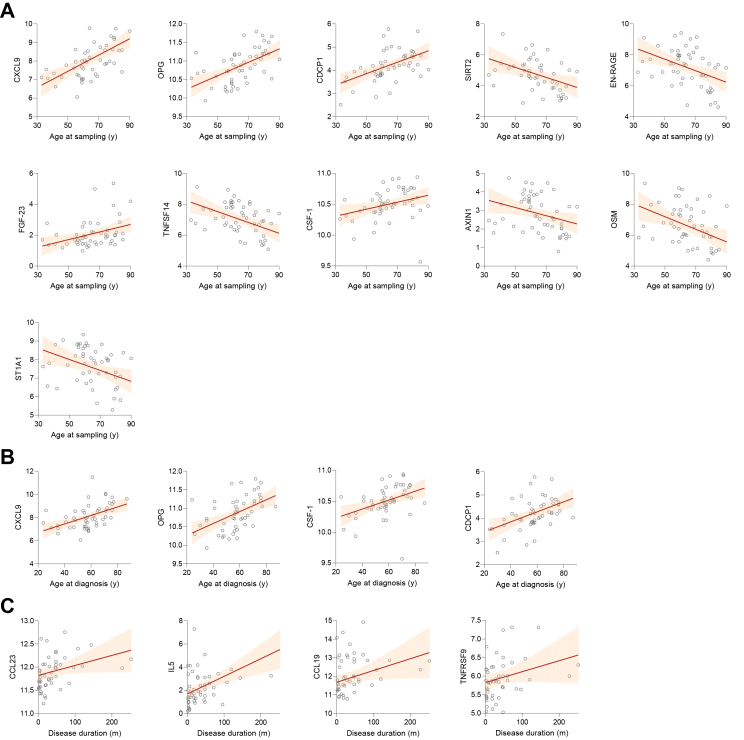
**Age and disease duration correlate with inflammatory protein signatures in CIDP.** (A) Age at sampling, (B) age at diagnosis, and (C) disease duration correlate with the expression levels of inflammatory proteins. Only proteins with a correlation coefficient (Spearman R) with moderate correlation values between 0.40 and 0.63 are represented. The red line with orange whiskers indicates a linear regression with mean and SEM. A full list of proteins with statistically significant correlation, including Spearman R and *p*-values, is provided in Supplementary Table S2.

In order to correlate clinical characteristics and subgroups of the CIDP cohort with inflammatory protein profile, we performed correlation analysis. Several inflammatory markers were significantly associated with age, muscle strength, and disability in CIDP. Age at both diagnosis and sampling correlated positively with CXCL9, CDCP1, OPG, and CSF-1. Notably, CXCL9 correlated with age at diagnosis (R = 0.56, *p* < 0.0001) and sampling (R = 0.63, *p* < 0.0001); CDCP1 with age at diagnosis (R = 0.48, *p* = 0.0004) and at sampling (R = 0.54, *p* < 0.0001) and similar robust associations were observed for OPG and CSF-1 (Fig. 3A and B, Supplementary Table S2).

**Supplementary Fig. S3:**
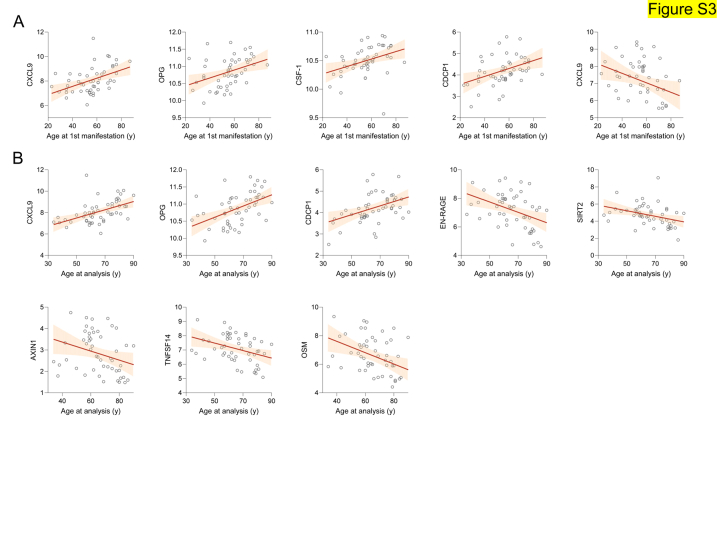
**Fig S3:** Correlations between inflammatory proteins and (A) age at first manifestation of the disease, and (B) age at analysis. Only proteins with a correlation coefficient (Spearman R) with moderate correlation values between 0.40–0.56 are represented.

Additional age-related associations at sampling included SIRT2, EN-RAGE, FGF-23, TNFSF14, CSF-1, AXIN1, OSM, and ST1A1 (Fig. 3A). Comparable patterns were seen for age at symptom onset, age at sampling (Supplementary Fig. S3, Supplementary Table S2), and for disease duration (Fig. 3C, Supplementary Table S2).

**Fig. 4:**
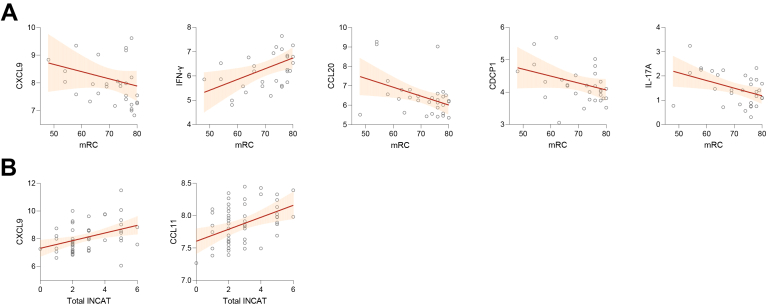
**Muscle strength and disease disability scores correlate with inflammatory proteins.** (A) mRC and (B) INCAT score correlate with the expression levels of inflammatory proteins. Only proteins with a correlation coefficient (Spearman R) with moderate correlation values between 0.40 and 0.47 are represented. A full list of proteins with statistically significant correlation, including Spearman R and *p*-values, is provided in Supplementary Table S2.

Muscle strength, assessed by MRC-sum score, showed moderate inverse correlations with CCL20 (R = −0.41; *p* = 0.020), CXCL9 (R = −0.44; *p* = 0.013), CDCP1 (R = −0.41; *p* = 0.021), and moderate positive correlation with IFN-γ (R = 0.41; *p* = 0.018; Fig. 4A). Conversely, disability measured by INCAT correlated positively with CXCL9 (R = 0.40; *p* = 0.0033), and CCL11 (R = 0.40; *p* = 0.0065; Fig. 4B).

### Calprotectin, neurofilament light chain, and cerebrospinal fluid protein levels associate with inflammatory serum markers

**Fig. 5:**
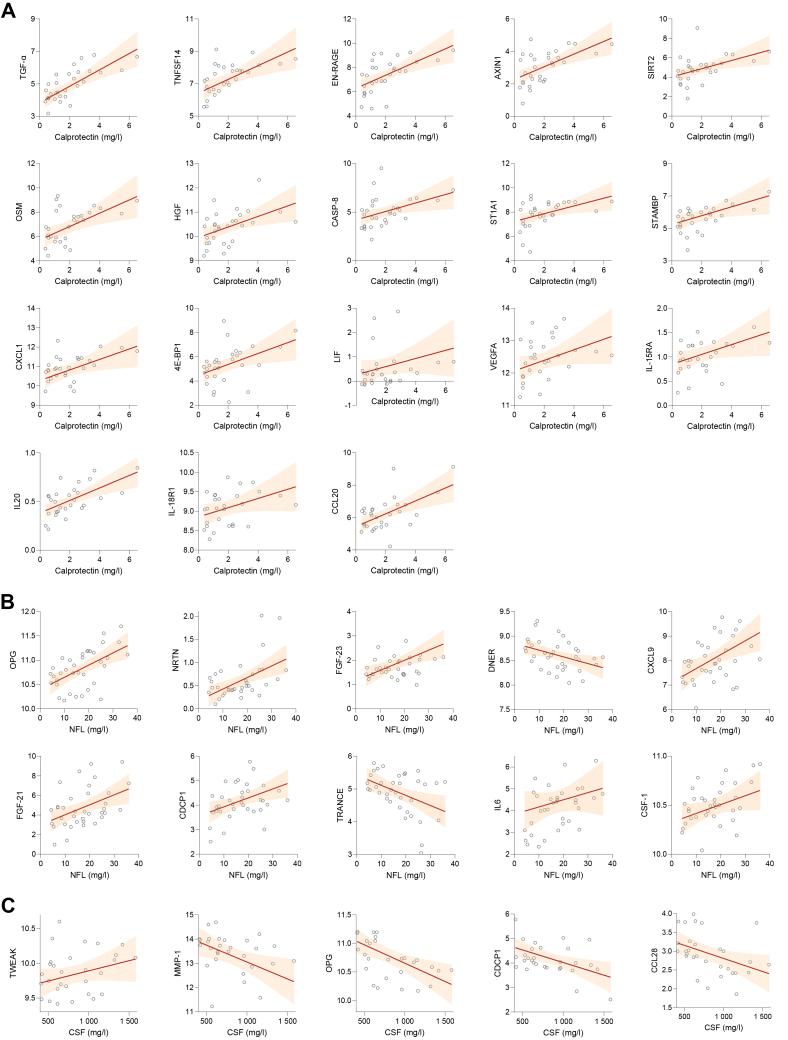
**Serum CLP, sNfL, and CSF protein correlate with inflammatory proteins.** (A) CLP, (B) NFL, and (C) CSF correlate with the expression levels of inflammatory proteins. Only proteins with a correlation coefficient (Spearman R) with moderate to strong correlation values between 0.40 and 0.73 are represented. A full list of proteins with statistically significant correlation, including Spearman R and *p*-values, is provided in Supplementary Table S2.

We next examined correlations between sCLP and inflammatory proteins, given prior reports linking elevated sCLP to active CIDP. Median sCLP was 1.5 pg/ml (IQR: 0.95–2.6). sCLP exhibited strong positive correlations with TGF-α (R = 0.73, *p* < 0.0001) and moderate positive correlations with TNFSF14 (R = 0.68, *p* < 0.0001), EN-RAGE (R = 0.65, *p* < 0.0001), AXIN1 (R = 0.59, *p* = 0.0006), SIRT2 (R = 0.56, *p* = 0.0014), CASP-8 (R = 0.52, *p* = 0.0033), ST1A1 (R = 0.52, *p* = 0.0035), OSM (R = 0.55, *p* = 0.0017), STAMBP (R = 0.52, *p* = 0.0036), CXCL1 (R = 0.51, *p* = 0.0037), and 4E-BP1 (R = 0.50, *p* = 0.0048; Fig. 5A, Supplementary Table S2).

sNfl (median 17.5 pg/ml, IQR: 9.6–25.0) correlated moderately with OPG (R = 0.58, *p* = 0.0002), FGF-21 (R = 0.45, *p* = 0.0053), FGF-23 (R = 0.48, *p* = 0.0028), NRTN (R = 0.49, *p* = 0.0020), CDCP1 (R = −0.42, *p* = 0.0098) and CXCL9 (R = −0.45, *p* = 0.0047; Fig. 5B), while DNER (R = −0.46, *p* = 0.0039), and TRANCE (R = −0.42, *p* = 0.010) had moderate inverse correlations.

CSF protein levels correlated with moderate inverse associations with MMP-1 (R = −0.46, *p* = 0.0062), OPG (R = −0.40, *p* = 0.019), CDCP1 (R = −0.40, *p* = 0.021), CCL28 (R = −0.40, *p* = 0.019), and a positive correlation for TWEAK (R = 0.43, *p* = 0.012; Fig. 5C).

### Serum immune signatures vary by sex, electrophysiology, disease stability, and treatment status

**Fig. 6:**
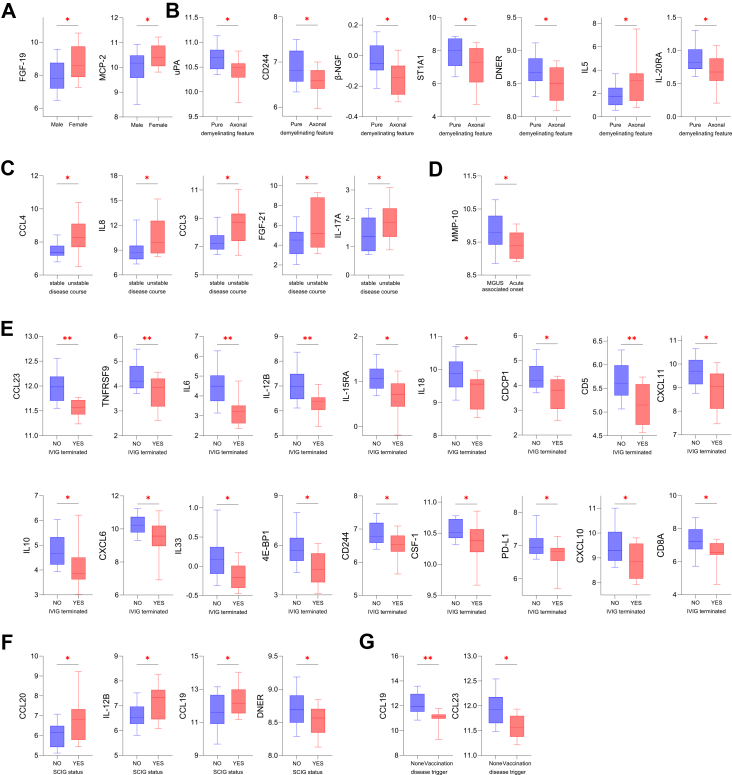
**Inflammatory protein expression differs in clinical subgroups of patients with CIDP based on (A) sex, (B) electrophysiological subtype, (C) disease stability, (D) disease form, (E) IVIg treatment, (F) SCIg treatment, and (G) disease trigger.** Data shown are NPX values plotted as a Box–Whiskers plot with median and 10th–90th percentile, and analysed with logistic regression Model 0, unadjusted for age and sex, presented in Supplementary Table S3. Only statistically significant changes between subgroups are shown, ∗*p* < 0.05, ∗∗*p* < 0.01.

The next aim was to perform exploratory analysis among multiple CIDP subgroups based on sex, electrophysiological pattern, disease stability, and immunotherapy status. Several inflammatory serum proteins differed across multiple clinical CIDP subgroups. Female patients had higher levels of FGF-19 (*p* = 0.042) and MCP-2 (*p* = 0.045; Fig. 6A, Supplementary Table S3).

Electrophysiological subtypes also displayed distinct signatures. Patients with pure demyelinating polyneuropathy had higher levels of uPA (*p* = 0.016), CD244 (*p* = 0.020), β-NGF (*p* = 0.026), ST1A1 (*p* = 0.028), DNER (*p* = 0.031), and IL20-RA (*p* = 0.044), and lower IL5 (*p* = 0.031; Fig. 6B, Supplementary Table S3) compared with those with axonal demyelinating CIDP.

Patients with unstable active disease showed elevated levels of CCL4 (*p* = 0.016), IL8 (*p* = 0.025), CCL3 (*p* = 0.026), FGF-21 (*p* = 0.029), and IL17A (*p* = 0.039) relative to those with stable active disease (Fig. 6C, Supplementary Table S3). In the onset subgroups, those with monoclonal gammopathy of undetermined significance (MGUS)-associated CIDP had higher MMP-10 levels (*p* = 0.023) than those with acute-onset CIDP (Fig. 6D, Supplementary Table S3).

Treatment status had a major impact on the immune profiles. Patients continuing IVIG treatment (n = 40) had higher levels of 18 inflammatory proteins, CCL23, TNFRSF9, IL6, IL-12B, IL15-RA, IL18, CDCP1, CD5, CXCL11, IL10, CXCL6, IL33, 4E-BP1, CD244, CSF-1, PD-L1, CXCL10, and CD8A compared with patients who had discontinued IVIG (n = 11, Fig. 6E, Supplementary Table S3). Patients receiving both IVIG and SCIG (n = 22) had higher concentrations of CCL20 (*p* = 0.026), IL-12B (*p* = 0.040), and CCL19 (*p* = 0.045) than those on IVIG alone (Fig. 6F, Supplementary Table S3), while DNER was higher in the IVIG-only group (*p* = 0.046).

Patients reporting a recent vaccination before symptom onset (n = 7) had lower levels of CCL19 (*p* = 0.021) and CCL23 (*p* = 0.042) compared with patients without identifiable triggers (n = 42, Fig. 6G).

### Serum IL8, EN-RAGE, and CASP-8 predicted improvement in CIDP

**Fig. 7:**
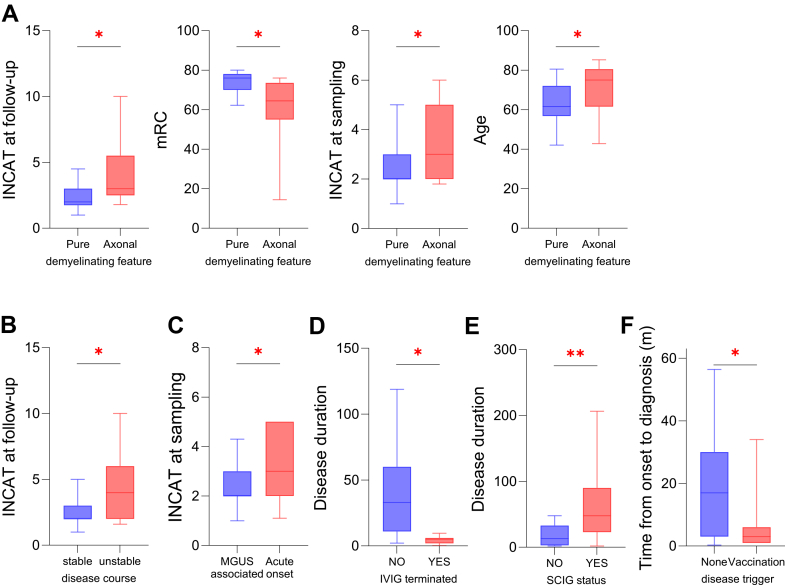
**Changes in the disease disability scores in the clinical subgroups of patients with CIDP that were classified based on (A) electrophysiological subtype, (B) disease stability, (C) disease form, (D) IVIg treatment, (E) SCIg treatment, and (F) disease trigger.** Data plotted as a Box–Whiskers plot with median and 10th–90th percentile, and analysed with logistic regression Model 0, unadjusted for age and sex, presented in Supplementary Table S3. Only statistically significant changes between subgroups are shown, ∗*p* < 0.05, ∗∗*p* < 0.01.

Further, we aimed to find out whether inflammatory signatures would predict clinical improvement of CIDP. Electrophysiological subtypes were associated with clinical severity: patients with axonal-demyelinating patterns on electroneurography had higher INCAT scores and lower MRC at both sampling and follow-up than those with pure demyelinating patterns (Fig. 7A). Higher INCAT scores were also observed in unstable disease and acute-onset CIDP (Fig. 7B and C). Patients on continuous IG therapy had longer disease duration (Fig. 7D and E), and those without an apparent trigger before disease onset experienced longer diagnostic delays (Fig. 7F).

**Fig. 8:**
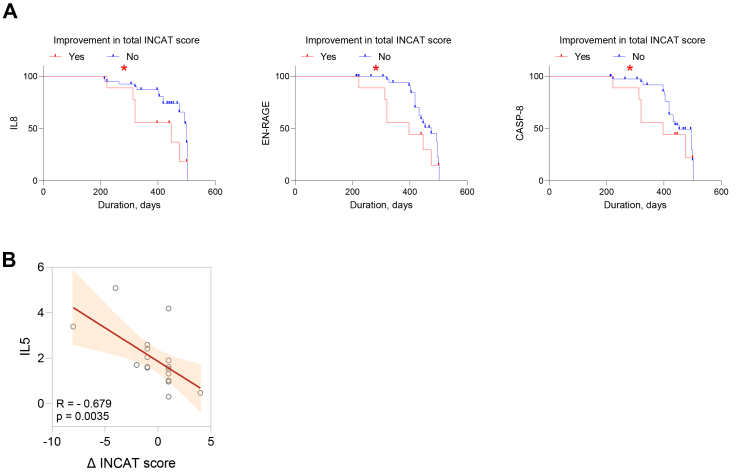
**Serum IL8, EN-RAGE, and CASP-8 as potential predictive biomarkers for clinical improvement in CIDP.** (A) Kaplan–Meier analysis predicting IL8, EN-RAGE, and CASP-8 levels as predictors of improvement (at least 1 point decrease in INCAT score) over time (from sampling to follow-up) in patients with CIDP. (B) Spearman correlation between serum IL5 levels and change in INCAT from sampling until follow-up.

In terms of potential predictors of clinical improvement, Kaplan–Meier analysis revealed that higher serum levels of IL8, EN-RAGE, and CASP-8 partially predicted clinical improvement, defined as a ≥1-point decrease in INCAT score from sampling to follow-up (Fig. 8A). Additionally, IL5 levels correlated with the magnitude of improvement (Fig. 8B), suggesting a potential utility in monitoring therapeutic response.

## Discussion

In this study, we used targeted proteomics to characterise inflammatory signatures in CIDP sera and to distinguish disease-specific processes from treatment-related effects. We identified a 13-protein signature that robustly differentiated CIDP from HC and neurological disease comparators, achieving high diagnostic performance with sensitivities and specificities up to 97% and 90%, respectively. This reproducible inflammatory pattern underscores a stable, systemic immune activation profile in CIDP and provides a foundation for biomarker-driven stratification. Building on this broader 13-protein signature, comparisons with treatment-naive MS and IVIG-treated MG enabled us to disentangle CIDP-specific inflammatory signatures from those influenced by IG therapy. Seven proteins, TNFSF14, CD40, SIRT2, CCL3, IL8, TGFα, and uPA, remained consistently elevated only in the CIDP group across all comparator groups, indicating that these markers are IG-independent and represent the most disease-specific components of the CIDP inflammatory signature. The inclusion of IVIG-treated patients with MG was particularly informative, as MG is a chronic antibody-mediated neuromuscular disorder that is sometimes managed with IVIG but lacks the demyelinating and axonal pathology characteristic of CIDP. This comparison enabled a partial assessment of immunoglobulin-related effects on inflammatory serum proteins. However, the observed differences between CIDP and MG likely also reflect disease-specific immunopathological mechanisms and should be interpreted in the context of the distinct treatment landscapes; patients with MG typically receive baseline immunotherapy with steroids and/or steroid-sparing agents, whereas this is generally not the case in CIDP. Importantly, the observation that key CIDP-associated inflammatory signatures were not uniformly mirrored in the MG control group supports the notion that the identified serum profiles are not merely driven by IVIG exposure but reflect CIDP-specific immune and neuroinflammatory processes. In contrast, six proteins, STAMBP, CASP-8, EN-RAGE, OSM, HGF, and CXCL6, were clearly influenced by IG-treatment, highlighting the importance of considering therapeutic context when interpreting serum biomarkers in CIDP.

The therapeutic landscape for CIDP is undergoing rapid transformation, with emerging treatments such as FcRn-inhibitors^11^ and complement-inhibitors like riliprubart (NCT06290141),^12^ empasiprubart (NCT06742190),^13^ and DNTH103 (NCT06858579) being investigated in phase III trials. This evolution reflects an urgent clinical need, as 10–15% of patients respond inadequately to standard-of-care therapies, and continue to experience long-term disability.^35^ The development of emerging targeted therapies in CIDP highlights the need for objective, biologically grounded biomarkers to guide treatment decisions, predict disease progression, and enable precision medicine. In this context, an important finding in our study is the identification of IL8, EN-RAGE, and CASP-8 as potential predictors of clinical improvement, defined by a reduction in INCAT score over time. Although the effect sizes were modest, the biological plausibility of these proteins, each linked to inflammatory signalling, myeloid activation, or apoptotic regulation, enhances the interpretative value of the associations. Nevertheless, these findings should be viewed as preliminary, and confirmation in larger, independent multicenter cohorts will be essential to establish their robustness and clinical utility.

IL8 is a potent neutrophil chemoattractant, reflecting active but potentially reversible inflammation, that has been found to be upregulated in the CSF of patients with CIDP compared to HCs.^26^ EN-RAGE (S100A12) is an established marker of systemic inflammation and was recently found to be lower in patients with MG receiving newer biologicals than in those receiving standard of care.^24^ CASP-8 has a role in apoptosis and inflammatory cell death signalling,^36^ possibly marking pathogenic but treatable immune activation in autoimmune demyelination.

Despite increasing interest in biological markers for CIDP, no serum or CSF biomarker has yet achieved widespread clinical implementation. Existing candidate biomarkers, including serum free light chains,^15^ sNfL,^14^^,^^16^^,^^37^ sCLP,^16^ ET-1,^18^ and peripherin,^19^ each offer valuable insights into immune activity or axonal damage but are limited by modest specificity or feasibility for routine use. Our findings extend this landscape by identifying inflammatory serum protein signatures that align closely with key clinical dimensions of CIDP, thereby supporting the potential for integrated biomarker strategies rather than reliance on a single analyte. Several inflammatory proteins, most notably CXCL9, CDCP1, OPG, and CSF-1, were positively associated with both age at diagnosis and age at sampling, suggesting an age-related upregulation of immune signatures. In contrast, SIRT2 and EN-RAGE had inverse associations with age, potentially reflecting regulatory mechanisms that decline with ageing. Together, these findings emphasise the importance of accounting for age-dependent immune alterations when interpreting serum biomarkers and may partially explain the clinical heterogeneity seen in CIDP.

sCLP, previously linked to active disease states,^16^ displayed broad correlations with inflammatory proteins involved in innate immunity and tissue remodelling, including TGF-α, TNFSF14, EN-RAGE, CASP-8, and CXCL1. This supports the use of sCLP not only as a standalone biomarker but also as a surrogate marker for the broader inflammatory milieu in CIDP, capturing both inflammatory and regenerative responses. Thus, therapeutic strategies aimed at modulating systemic inflammation may be clinically relevant.

The axonal injury-associated marker sNfL correlated positively with OPG, FGF-21, FGF-23, NRTN, CDCP1, and CXCL9, and inversely with DNER and TRANCE, indicating that axonal degeneration may arise through partially distinct immunometabolic pathways. These divergent correlations imply that neuroaxonal damage can progress independently of overt immune activation, highlighting the value of combined biomarkers for disease staging and therapeutic timing.

Finally, some inflammatory proteins also correlated with clinical measures of disability. CXCL9, CDCP1, CCL20, and OPG were inversely associated with muscle strength (MRC-sum scores) and positively associated with INCAT disability scores, suggesting utility as dynamic biomarkers of disease activity. Additionally, serum inflammatory proteins, such as MMP-1 and TWEAK, showed moderate correlations with CSF protein levels, suggesting that selected serum markers may reflect intrathecal immune activity and offer an accessible window into processes typically assessed by lumbar puncture.

Although individual proteins showed variable associations with specific clinical parameters, several markers converge on broader biological processes relevant to CIDP pathophysiology. For example, chemokine-mediated leucocyte recruitment pathways are reflected by markers such as IL8, CCL3, and CXCL9,^23^^,^^38^ while proteins including TNFSF14 and EN-RAGE (S100A12) are linked to innate immune activation and inflammatory signalling.^39^^,^^40^ CASP-8 may indicate apoptosis-related inflammatory pathways associated with tissue injury.^41^ Importantly, overlapping associations between inflammatory proteins, serum calprotectin, and neurofilament light chain suggest that systemic immune activation and neuroaxonal injury represent interconnected but partially distinct biological processes in CIDP.

Most patients were receiving immunomodulatory treatment at the time of sampling, which may have influenced protein levels; however, this reflects *real-world* clinical practice and is relevant for identifying biomarkers useful for disease monitoring. Including an IVIG-treated MG control group helped reduce confounding by IG therapy and partially separated treatment-related signatures from CIDP-specific inflammatory signatures. Disease activity in this study was primarily classified using the CIDP Disease Activity Scale (CDAS), which distinguishes immunologically active disease from residual neurological impairment based on clinical course and treatment dependency. However, CDAS categories—particularly “active stable disease”—may encompass heterogeneous clinical scenarios and may not fully capture short-term disease fluctuations. Follow-up clinical data, including changes in INCAT disability scores after sampling, were available for a subset of patients and provided additional clinical context for interpreting disease dynamics and treatment-related changes. Future prospective studies with standardised longitudinal clinical and biomarker assessments will be important to further refine biomarker associations with disease activity.

The focus on typical CIDP may limit the generalisability of our findings to atypical variants, particularly as patients with IgG4 paranodal antibodies were not included. Moreover, the cross-sectional design precludes conclusions about temporal dynamics or causality. The sample size was modest, and the targeted proteomic panel may have missed relevant non-inflammatory pathways of relevance. Although age and sex were included as covariates in the primary regression analyses, residual confounding effects due to age-related inflammatory drift cannot be fully excluded. Finally, both the correlation analyses and the subgroup findings should be regarded as exploratory and primarily hypothesis-generating. Despite these limitations, the study has notable strengths. We applied a robust proteomic approach to a well-characterised CIDP cohort, identified immune signatures associated with disease activity, age, and axonal injury, and demonstrated clear associations with clinical disability. Together, these findings support the utility of serum immune profiling for disease monitoring, patient stratification, and improved clinical phenotyping in CIDP.

In conclusion, our study demonstrates that CIDP is characterised by a distinct and systemic inflammatory signature. Several proteins, including CXCL9, CDCP1, and sCLP, consistently correlated with clinical measures of disease activity, while others reflected age-dependent immune variation, disease severity, or axonal injury. By incorporating an IVIG-treated MG control, we were able to separate CIDP-specific signatures from treatment-modulated inflammatory signatures. Together, these findings highlight the potential of serum immune profiling as a valuable tool for patient stratification, disease monitoring, and individualised therapeutic decision-making, an approach that aligns with the rapidly evolving therapeutic landscape of CIDP.

## Contributors

**Amol K. Bhandage**: Conceptualisation, Data curation, Formal analysis, Investigation, Methodology, Visualisation, Writing—review and editing.

**Frauke Stascheit**: Conceptualisation, Data curation, Formal analysis, Investigation, Methodology, Visualisation, Writing—review and editing.

**Hannah Preßler:** Investigation, Writing—review and editing.

**Sarah Hoffmann:** Methodology, Resources, Writing—review and editing.

**Andreas Meisel**: Methodology, Resources, Funding acquisition, Writing—review and editing.

**Anna Rostedt Punga**: Conceptualisation, Investigation, Funding acquisition, Project administration, Resources, Supervision, Writing—original draft, Writing—review and editing.

All authors have read and approved the final version of the manuscript. Amol K. Bhandage, Frauke Stascheit, and Anna Rostedt Punga verified the underlying data.

## Data sharing statement

All data supporting the findings of this study are available within the article and its Supplementary Information. The raw proteomic data generated using the Olink® Target 96 Inflammation panel, along with associated sample metadata, are available from the corresponding author upon reasonable request. However, due to restrictions on informed consent, individual-level data can only be shared under a data-sharing agreement that ensures appropriate protection of participant confidentiality. This study did not generate any new unique reagents or custom code.

## Declaration of interests

F.S. received travel/accommodation/meeting expenses from Alexion Pharmaceuticals and argenx, and received speaking honoraria and honoraria for attendance at advisory boards from Alexion Pharmaceuticals, argenx, and UCB Pharma. She receives financial research support (paid to her institution) from Alexion Pharmaceuticals, argenx, and Cytel, and serves on the medical advisory board of the German Myasthenia Gravis Society. A.R.P. has received consultancy fees from argenx, UCB, Dianthus, Alexion, Novartis, and Toleranzia, which are unrelated to this study. S.H. has received speaker’s honoraria, consulting fees, or (institutional) financial research support from Alexion, argenx, UCB, Grifols, Roche, Novartis, and Johnson&Johnson. She serves on the medical advisory board of the German MG Society. A.M. received speaker or consultancy honoraria or financial research support (paid to his institution) from Alexion Pharmaceuticals, argenx, Amgen, Axunio, Desitin, Grifols, Janssen, Merck, Novartis, Octapharma, Sanofi, and UCB, and serves as a member of the medical advisory board of the German Myasthenia Gravis Society and chairman of the *Association for Research in Myasthenic Syndromes in Germany*. Other authors declare no conflicts of interest.
