# Supplementary material for: Serum inflammatory proteomic signatures define chronic inflammatory demyelinating polyneuropathy and inform on disease activity
Source: eBioMedicine. 2026 Jun 25;129:106348. doi: 10.1016/j.ebiom.2026.106348 (PMC13325459; doi:10.1016/j.ebiom.2026.106348)
Supplement: Supplementary Table S1 [file mmc1.pdf]

Table S1: Logistic regression analysis with Model 0, unadjusted and Model 1, adjusted for age and sex, for the comparison of CIDP vs MS/HC for all proteins.

CIDP vs MS	Model 0					Model 1					Signi. in		
Protein	OR	95% CI		P-value	C-stat	OR	95% CI		P-value	C-stat	Model 0 + 1	Boruta	All
ST1A1	4.20	2.75	6.44	3.99E-11	0.910	5.19	2.99	9.03	5.28E-09	0.981	Yes	Yes	Yes
TNFSF14	5.61	3.33	9.45	9.27E-11	0.915	6.61	3.42	12.79	2.04E-08	0.978	Yes	Yes	Yes
CD40	35.54	11.71	107.87	2.9E-10	0.919	20.50	6.21	67.64	7.13E-07	0.949	Yes	Yes	Yes
SIRT2	4.04	2.57	6.34	1.5E-09	0.871	5.18	2.93	9.14	1.39E-08	0.968	Yes	Yes	Yes
AXIN1	4.39	2.70	7.13	2.49E-09	0.871	5.17	2.85	9.38	6.81E-08	0.958	Yes	Yes	Yes
STAMBP	5.82	3.23	10.52	5.03E-09	0.863	7.24	3.54	14.81	5.85E-08	0.956	Yes	Yes	Yes
IL7	0.05	0.02	0.14	5.59E-09	0.843	0.07	0.02	0.20	1.55E-06	0.931	Yes	Yes	Yes
CASP8	3.29	2.15	5.03	3.83E-08	0.815	3.28	2.00	5.40	2.81E-06	0.936	Yes	Yes	Yes
CDCP1	5.86	3.06	11.23	9.63E-08	0.838	2.07	0.99	4.34	0.0541	0.878		Yes	
ENRAGE	2.80	1.89	4.14	2.91E-07	0.776	3.99	2.32	6.86	5.56E-07	0.945	Yes	Yes	Yes
CCL3	3.85	2.29	6.47	3.49E-07	0.829	2.58	1.52	4.40	0.0005	0.915	Yes	Yes	Yes
IL8	3.67	2.21	6.11	5.4E-07	0.837	2.92	1.73	4.95	6.6E-05	0.938	Yes	Yes	Yes
MCP1	7.18	3.25	15.86	1.11E-06	0.788	5.75	2.19	15.06	0.0004	0.910	Yes	Yes	Yes
IL18	5.36	2.72	10.55	1.21E-06	0.791	2.60	1.24	5.47	0.0115	0.883		Yes	
OPG	8.95	3.66	21.89	1.59E-06	0.771	3.16	1.09	9.14	0.0339	0.879		Yes	
TGFa	2.68	1.78	4.03	2.36E-06	0.773	3.40	2.01	5.74	4.76E-06	0.931	Yes	Yes	Yes
MCP4	4.71	2.47	8.98	2.48E-06	0.777	3.75	1.76	7.97	0.0006	0.900		Yes	
Flt3L	6.22	2.87	13.48	3.63E-06	0.770	2.23	0.90	5.53	0.0828	0.876		Yes	
OSM	2.23	1.59	3.14	3.87E-06	0.756	2.70	1.75	4.15	6.91E-06	0.930	Yes	Yes	Yes
ADA	7.25	3.07	17.14	6.39E-06	0.762	11.28	3.76	33.86	1.55E-05	0.921	Yes	Yes	Yes
CST5	4.10	2.18	7.74	1.3E-05	0.731	2.17	1.01	4.70	0.0480	0.876			
CCL11	8.52	3.25	22.36	1.34E-05	0.764	3.89	1.28	11.76	0.0164	0.883		Yes	
IL10	2.89	1.78	4.68	1.63E-05	0.765	2.28	1.34	3.90	0.0025	0.887		Yes	
CXCL10	2.69	1.71	4.21	1.66E-05	0.767	1.60	0.95	2.67	0.0749	0.878		Yes	
CCL4	3.16	1.85	5.40	2.39E-05	0.721	2.22	1.18	4.21	0.0140	0.888		Yes	
IL6	2.10	1.48	2.98	3.58E-05	0.750	1.58	1.12	2.24	0.0100	0.890			
uPA	9.93	3.29	30.00	4.7E-05	0.739	15.38	3.99	59.30	7.2E-05	0.910	Yes		
HGF	3.72	1.93	7.16	8.4E-05	0.714	5.33	2.16	13.19	0.0003	0.912	Yes		
CSF1	17.33	4.06	73.95	0.0001	0.718	3.75	0.69	20.52	0.1269	0.877			
FGF23	3.21	1.77	5.81	0.0001	0.780	2.85	1.36	5.99	0.0056	0.892		Yes	
FGF21	1.53	1.22	1.90	0.0002	0.691	1.17	0.90	1.52	0.2434	0.871			
TNFRSF9	3.46	1.80	6.66	0.0002	0.702	1.96	0.86	4.45	0.1070	0.877			
VEGFA	2.95	1.66	5.23	0.0002	0.699	2.45	1.24	4.85	0.0096	0.892			
CXCL11	2.25	1.46	3.47	0.0003	0.732	1.94	1.16	3.26	0.0115	0.890			
CXCL6	2.80	1.61	4.88	0.0003	0.710	4.50	2.21	9.15	3.36E-05	0.923	Yes		
CD8A	0.42	0.26	0.67	0.0003	0.690	0.57	0.32	1.01	0.0527	0.880		Yes	
CXCL9	1.90	1.32	2.73	0.0005	0.713	0.85	0.55	1.31	0.4694	0.873			
MCP3	2.02	1.33	3.06	0.0009	0.689	1.52	1.06	2.17	0.0226	0.886			
IL15RA	4.48	1.74	11.55	0.0019	0.694	1.47	0.50	4.34	0.4825	0.870			
IL5	1.47	1.15	1.89	0.0020	0.698	1.38	1.02	1.86	0.0339	0.885			
IL10RB	4.84	1.78	13.19	0.0020	0.678	1.66	0.49	5.58	0.4119	0.873			
PDL1	3.92	1.61	9.55	0.0027	0.654	1.21	0.41	3.59	0.7350	0.870			
FGF5	9.14	2.15	38.76	0.0027	0.680	3.71	0.73	18.91	0.1149	0.873		Yes	
LIF	2.73	1.39	5.38	0.0036	0.678	3.99	1.68	9.49	0.0017	0.904			
IL18R1	2.92	1.41	6.03	0.0038	0.646	2.27	0.92	5.61	0.0759	0.875			
TNFa	2.01	1.20	3.37	0.0084	0.740	1.64	1.04	2.58	0.0319	0.878			
LIFR	4.42	1.38	14.13	0.0122	0.628	4.31	0.98	18.86	0.0525	0.879			
CD6	2.13	1.15	3.94	0.0163	0.615	2.22	1.06	4.67	0.0349	0.880			
CXCL5	0.61	0.41	0.91	0.0165	0.619	0.93	0.57	1.52	0.7745	0.871		Yes	
CD5	2.52	1.17	5.44	0.0181	0.622	2.22	0.85	5.77	0.1015	0.878			
CCL19	1.51	1.07	2.15	0.0199	0.643	1.18	0.79	1.74	0.4177	0.871			
CCL20	1.50	1.06	2.13	0.0206	0.613	1.39	0.93	2.09	0.1094	0.872			
NRTN	2.26	1.08	4.73	0.0312	0.645	1.92	0.80	4.63	0.1436	0.874			
TRANCE	0.60	0.37	0.96	0.0326	0.598	0.75	0.42	1.34	0.3289	0.873			
CXCL1	1.79	1.03	3.12	0.0387	0.652	2.78	1.45	5.36	0.0022	0.893			
SCF	0.47	0.23	0.96	0.0394	0.565	0.70	0.29	1.67	0.4182	0.871			
CCL25	1.75	1.01	3.03	0.0448	0.613	0.87	0.41	1.84	0.7202	0.872			
SLAMF1	1.87	1.00	3.47	0.0486	0.599	1.22	0.55	2.69	0.6285	0.870			
IL17C	1.60	0.99	2.61	0.0573	0.579	1.11	0.60	2.07	0.7447	0.872			

CIDP vs MS	Model 0						Model 1						Signi. in		
Protein	OR	95% CI		P-value	C-stat		OR	95% CI		P-value	C-stat		Model 0 + 1	Boruta	All
IL33	2.06	0.97	4.36	0.0583	0.609		3.40	1.30	8.94	0.0129	0.882				
CX3CL1	1.96	0.96	4.00	0.0643	0.614		1.13	0.44	2.88	0.8056	0.871				
MMP10	1.55	0.94	2.55	0.0862	0.590		1.31	0.69	2.48	0.4132	0.869				
CCL23	0.50	0.23	1.11	0.0881	0.626		0.38	0.14	1.02	0.0557	0.882				
IL17A	1.44	0.91	2.27	0.1225	0.592		1.51	0.87	2.63	0.1406	0.874				
GDNF	0.50	0.20	1.21	0.1244	0.596		0.28	0.08	0.92	0.0355	0.884				
CCL28	0.72	0.47	1.11	0.1375	0.565		0.64	0.36	1.14	0.1308	0.879				
IL1a	0.70	0.44	1.14	0.1508	0.549		0.70	0.40	1.25	0.2273	0.874				
4EBP1	1.19	0.92	1.54	0.1798	0.563		1.15	0.83	1.60	0.3912	0.873				
IL12B	1.34	0.86	2.07	0.1923	0.542		1.27	0.73	2.22	0.4039	0.869				
MMP1	1.26	0.87	1.82	0.2188	0.580		1.18	0.77	1.81	0.4556	0.872				
IL4	1.28	0.86	1.91	0.2250	0.583		1.44	0.83	2.49	0.1937	0.873				
MCP2	1.30	0.79	2.12	0.2997	0.570		1.53	0.80	2.91	0.1961	0.875				
IL22RA1	1.22	0.79	1.88	0.3669	0.524		1.23	0.72	2.11	0.4500	0.872				
IL24	0.81	0.51	1.30	0.3799	0.533		1.02	0.56	1.85	0.9467	0.871				
DNER	0.61	0.19	1.98	0.4081	0.552		1.71	0.39	7.51	0.4757	0.871				
IL20	1.47	0.59	3.66	0.4088	0.563		1.16	0.38	3.51	0.7925	0.872				
NT3	0.76	0.39	1.47	0.4148	0.616		2.04	0.79	5.23	0.1386	0.877				
CD244	1.39	0.62	3.14	0.4224	0.515		2.32	0.77	7.00	0.1345	0.874				
IL2RB	0.73	0.33	1.60	0.4360	0.516		0.70	0.26	1.88	0.4767	0.872				
TSLP	0.85	0.57	1.28	0.4480	0.545		0.84	0.51	1.38	0.4808	0.872				
ARTN	0.81	0.46	1.43	0.4659	0.498		0.70	0.34	1.47	0.3501	0.875				
IL10RA	0.80	0.43	1.47	0.4692	0.544		0.74	0.33	1.65	0.4657	0.871				
IL13	1.13	0.74	1.72	0.5707	0.485		1.35	0.81	2.25	0.2489	0.871				
TNFb	0.84	0.44	1.62	0.6042	0.513		0.98	0.41	2.32	0.9651	0.871				
TGFb1	1.29	0.48	3.48	0.6190	0.543		1.04	0.29	3.68	0.9530	0.871				
IL2	1.31	0.43	3.96	0.6350	0.529		1.52	0.37	6.23	0.5575	0.872				
FGF19	0.93	0.68	1.28	0.6546	0.531		0.84	0.55	1.28	0.4083	0.874				
bNGF	1.67	0.11	25.56	0.7110	0.524		1.67	0.06	43.17	0.7567	0.871				
IL20RA	1.13	0.46	2.78	0.7827	0.563		1.94	0.59	6.31	0.2733	0.874				
TRAIL	0.88	0.35	2.25	0.7968	0.504		0.47	0.12	1.76	0.2611	0.871				
IFNg	1.02	0.76	1.36	0.8907	0.512		0.70	0.48	1.03	0.0738	0.877				
TWEAK	1.01	0.42	2.41	0.9902	0.496		2.96	0.86	10.14	0.0843	0.879				

CIDP vs HC	Model 0					Model 1					Signi. in		
Protein	OR	95% CI		P-value	C-stat	OR	95% CI		P-value	C-stat	Model 0 + 1	Boruta	All
IL8	6.48	3.79	11.08	7.97E-12	0.906	5.63	3.24	9.80	9.37E-10	0.784	Yes	Yes	Yes
TGFa	6.53	3.78	11.28	1.81E-11	0.854	9.97	5.10	19.51	1.86E-11	0.765	Yes	Yes	Yes
IL6	4.62	2.95	7.22	2.04E-11	0.862	3.67	2.31	5.83	3.85E-08	0.762	Yes	Yes	Yes
OSM	3.51	2.36	5.21	5.9E-10	0.790	4.63	2.87	7.46	3.14E-10	0.765	Yes	Yes	Yes
TNFSF14	4.31	2.71	6.84	5.94E-10	0.801	4.83	2.94	7.92	4.54E-10	0.771	Yes	Yes	Yes
IL10	5.10	3.04	8.55	6.51E-10	0.835	4.50	2.66	7.61	1.94E-08	0.768	Yes	Yes	Yes
OPG	19.07	7.35	49.48	1.36E-09	0.784	10.17	3.43	30.16	2.89E-05	0.897	Yes	Yes	Yes
CDCP1	6.14	3.41	11.06	1.56E-09	0.811	4.20	2.19	8.06	1.56E-05	0.782	Yes	Yes	Yes
CCL3	4.83	2.89	8.05	1.66E-09	0.818	4.02	2.40	6.72	1.16E-07	0.805	Yes	Yes	Yes
CD40	16.21	6.45	40.74	3.16E-09	0.800	12.62	4.77	33.41	3.32E-07	0.796	Yes	Yes	Yes
CASP8	3.50	2.28	5.37	9.98E-09	0.739	3.68	2.32	5.84	3.4E-08	0.762	Yes	Yes	Yes
ENRAGE	2.83	1.92	4.19	1.78E-07	0.727	3.18	2.08	4.87	1E-07	0.772	Yes	Yes	Yes
FGF21	1.81	1.44	2.27	2.76E-07	0.719	1.62	1.26	2.07	0.0001	0.761	Yes	Yes	Yes
HGF	7.35	3.42	15.79	3.15E-07	0.719	9.46	3.86	23.15	8.72E-07	0.768	Yes	Yes	Yes
uPA	23.22	6.84	78.87	4.61E-07	0.755	21.50	5.73	80.71	5.44E-06	0.787	Yes	Yes	Yes
IL18	5.11	2.69	9.70	6.09E-07	0.732	4.12	2.07	8.18	5.22E-05	0.800	Yes	Yes	Yes
CCL4	3.14	1.95	5.06	2.47E-06	0.713	2.78	1.68	4.59	7.13E-05	0.764	Yes	Yes	Yes
CXCL10	2.50	1.68	3.73	7.18E-06	0.717	1.77	1.15	2.74	0.0099	0.770			
STAMBP	3.36	1.96	5.75	1.03E-05	0.720	3.57	1.99	6.41	2.03E-05	0.921	Yes	Yes	Yes
TNFRSF9	4.67	2.32	9.39	1.58E-05	0.696	3.04	1.42	6.51	0.0041	0.889			
ADA	4.99	2.26	10.99	6.65E-05	0.681	6.03	2.46	14.77	8.35E-05	0.763	Yes	Yes	Yes
CSF1	16.25	4.12	64.04	6.74E-05	0.700	7.07	1.60	31.29	0.0099	0.763			
CD5	4.63	2.16	9.93	8.12E-05	0.685	3.80	1.64	8.81	0.0018	0.777		Yes	
IL5	1.51	1.23	1.86	8.48E-05	0.733	1.65	1.31	2.08	2.03E-05	0.881	Yes	Yes	Yes
IL18R1	4.42	2.10	9.29	8.75E-05	0.689	4.49	1.92	10.50	0.0005	0.764	Yes		
SIRT2	1.85	1.35	2.54	0.0001	0.669	2.22	1.53	3.24	3.07E-05	0.762	Yes		
FGF5	15.85	3.81	65.91	0.0001	0.676	5.26	1.13	24.50	0.0343	0.763			
4EBP1	1.79	1.32	2.43	0.0002	0.675	1.60	1.17	2.20	0.0034	0.819			
CCL19	1.85	1.34	2.57	0.0002	0.693	1.70	1.21	2.40	0.0024	0.790		Yes	
CCL20	1.96	1.37	2.80	0.0002	0.650	1.77	1.22	2.56	0.0026	0.767			
LIFR	7.65	2.56	22.86	0.0003	0.665	5.93	1.79	19.59	0.0035	0.767			
TNFa	2.56	1.54	4.25	0.0003	0.727	2.35	1.44	3.82	0.0006	0.762			
IL10RB	7.15	2.46	20.81	0.0003	0.674	3.72	1.16	11.93	0.0268	0.776			
CXCL9	1.92	1.35	2.74	0.0003	0.642	1.12	0.73	1.72	0.6141	0.791			
CX3CL1	3.87	1.85	8.08	0.0003	0.672	2.78	1.25	6.18	0.0119	0.782			
PDL1	4.42	1.93	10.12	0.0004	0.663	2.54	1.05	6.17	0.0388	0.786			
MCP3	2.08	1.38	3.12	0.0004	0.587	1.72	1.21	2.45	0.0027	0.762			
FGF23	2.86	1.54	5.30	0.0009	0.570	1.80	0.93	3.48	0.0806	0.770		Yes	
VEGFA	2.48	1.43	4.30	0.0012	0.647	2.07	1.13	3.79	0.0189	0.792			
CD8A	0.51	0.34	0.77	0.0013	0.628	0.65	0.42	1.00	0.0483	0.819		Yes	
DNER	0.15	0.05	0.49	0.0015	0.642	0.13	0.04	0.51	0.0031	0.852			
CCL28	0.41	0.23	0.72	0.0018	0.662	0.32	0.17	0.61	0.0005	0.862		Yes	
CXCL6	2.14	1.31	3.51	0.0026	0.651	2.82	1.62	4.89	0.0002	0.760			
CST5	2.36	1.34	4.14	0.0029	0.624	1.44	0.77	2.71	0.2515	0.783			
TRANCE	0.50	0.32	0.79	0.0029	0.633	0.69	0.42	1.14	0.1524	0.763		Yes	
SCF	0.37	0.19	0.73	0.0036	0.594	0.39	0.19	0.79	0.0092	0.827			
CXCL11	1.95	1.24	3.06	0.0036	0.653	1.66	1.03	2.66	0.0370	0.762			
IL17A	1.77	1.17	2.66	0.0063	0.636	1.95	1.25	3.06	0.0033	0.765			
IL15RA	3.37	1.40	8.11	0.0068	0.632	1.51	0.59	3.86	0.3843	0.793			
MMP10	2.10	1.23	3.59	0.0069	0.603	1.68	0.92	3.05	0.0911	0.769			
CD6	2.12	1.22	3.68	0.0077	0.615	1.77	0.98	3.20	0.0605	0.767			
MCP1	2.29	1.24	4.22	0.0079	0.594	1.63	0.84	3.14	0.1470	0.762			
Flt3L	2.38	1.20	4.75	0.0135	0.610	1.12	0.52	2.45	0.7678	0.764		Yes	
CCL25	1.90	1.09	3.29	0.0225	0.598	1.08	0.59	1.99	0.8033	0.801			
LIF	1.56	1.06	2.29	0.0255	0.622	1.59	1.02	2.47	0.0400	0.784			
IL33	1.92	1.06	3.51	0.0327	0.637	2.03	1.13	3.64	0.0177	0.763			
CCL11	2.28	1.06	4.90	0.0351	0.599	1.02	0.43	2.43	0.9553	0.788			
TRAIL	0.38	0.16	0.94	0.0371	0.590	0.37	0.13	1.01	0.0523	0.765		Yes	
IL12B	1.55	0.98	2.44	0.0601	0.564	1.68	1.02	2.76	0.0421	0.770			

CIDP vs HC	Model 0					Model 1						Signi. in	
Protein	OR	95% CI		P-value	C-stat	OR	95% CI		P-value	C-stat	Model 0 + 1	Boruta	All
CXCL1	1.65	0.97	2.80	0.0632	0.632	1.81	1.07	3.08	0.0281	0.781			
IL2	2.80	0.91	8.60	0.0731	0.588	4.76	1.39	16.27	0.0129	0.783			
NRTN	1.57	0.96	2.59	0.0742	0.667	1.48	0.89	2.45	0.1279	0.770			
TGFb1	2.37	0.87	6.46	0.0901	0.603	1.84	0.64	5.27	0.2563	0.797			
SLAMF1	1.58	0.93	2.69	0.0912	0.576	1.07	0.60	1.92	0.8239	0.779			
IL13	1.38	0.93	2.04	0.1086	0.524	1.46	0.95	2.23	0.0851	0.763			
MCP4	1.46	0.91	2.32	0.1135	0.592	1.17	0.72	1.92	0.5214	0.761			
IL17C	1.36	0.90	2.06	0.1489	0.553	1.15	0.73	1.82	0.5547	0.821			
IL10RA	0.71	0.43	1.15	0.1651	0.550	0.73	0.42	1.25	0.2529	0.771			
IL20	1.68	0.78	3.63	0.1876	0.596	2.24	0.96	5.27	0.0637	0.766			
NT3	1.32	0.84	2.08	0.2256	0.534	1.50	0.92	2.42	0.1006	0.804			
FGF19	0.83	0.61	1.13	0.2291	0.554	0.82	0.59	1.15	0.2445	0.800			
ST1A1	1.19	0.89	1.59	0.2360	0.563	1.33	0.97	1.82	0.0803	0.832			
bNGF	0.26	0.02	3.26	0.2944	0.535	0.14	0.01	2.13	0.1568	0.864			
CXCL5	0.84	0.60	1.18	0.3241	0.523	0.99	0.68	1.43	0.9390	0.807			
CCL23	0.68	0.31	1.48	0.3326	0.580	0.61	0.27	1.39	0.2398	0.762		Yes	
IL22RA1	1.19	0.78	1.80	0.4231	0.520	1.22	0.78	1.91	0.3773	0.764			
IL1a	0.82	0.50	1.35	0.4408	0.534	0.88	0.52	1.48	0.6304	0.790			
CD244	0.81	0.34	1.92	0.6305	0.552	0.95	0.37	2.45	0.9232	0.849		Yes	
MCP2	1.13	0.69	1.83	0.6323	0.534	1.16	0.69	1.95	0.5781	0.788			
IL2RB	1.21	0.54	2.72	0.6371	0.515	1.08	0.45	2.60	0.8687	0.783			
IL20RA	0.86	0.45	1.67	0.6628	0.471	0.69	0.30	1.55	0.3664	0.759			
MMP1	1.08	0.69	1.68	0.7423	0.550	1.07	0.68	1.68	0.7713	0.768			
IL4	1.06	0.74	1.50	0.7619	0.512	1.10	0.76	1.60	0.6060	0.819			
IL7	1.09	0.57	2.06	0.7975	0.514	1.00	0.50	2.01	0.9896	0.929			
IFNg	0.96	0.71	1.30	0.8099	0.513	0.80	0.56	1.14	0.2253	0.860			
AXIN1	0.96	0.64	1.45	0.8463	0.518	1.19	0.77	1.84	0.4413	0.768		Yes	
TWEAK	1.06	0.44	2.55	0.9013	0.510	1.54	0.56	4.25	0.4005	0.831			
TSLP	1.01	0.69	1.50	0.9416	0.506	0.96	0.63	1.46	0.8464	0.762			
GDNF	1.02	0.52	2.02	0.9531	0.490	0.65	0.28	1.50	0.3078	0.843			
IL24	1.01	0.66	1.54	0.9703	0.503	1.05	0.67	1.65	0.8380	0.763			
ARTN	0.99	0.57	1.74	0.9785	0.477	0.95	0.48	1.88	0.8733	0.770			
TNFb	0.99	0.50	1.98	0.9801	0.490	1.25	0.57	2.75	0.5754	0.788			
