# Supplementary material for: Serum inflammatory proteomic signatures define chronic inflammatory demyelinating polyneuropathy and inform on disease activity
Source: eBioMedicine. 2026 Jun 25;129:106348. doi: 10.1016/j.ebiom.2026.106348 (PMC13325459; doi:10.1016/j.ebiom.2026.106348)
Supplement: Supplementary Table S2 [file mmc2.pdf]

Table S2: Spearman correlation between inflammatory proteins expression levels in serum and the clinical features of the CIDP patients

Related to figure 2	Protein	Spearman r	95% CI	p value	Significance	# XY Pairs
Age at sampling (y)	CXCL9	0.63	0.42, 0.78	<0.0001	****	51
Age at sampling (y)	OPG	0.60	0.38, 0.75	<0.0001	****	51
Age at sampling (y)	CDCP1	0.54	0.30, 0.71	<0.0001	****	51
Age at sampling (y)	SIRT2	-0.43	-0.64, -0.17	0.0016	**	51
Age at sampling (y)	EN-RAGE	-0.43	-0.63, -0.16	0.0018	**	51
Age at sampling (y)	FGF-23	0.42	0.16, 0.63	0.0019	**	51
Age at sampling (y)	TNFSF14	-0.42	-0.63, -0.15	0.0022	**	51
Age at sampling (y)	CSF-1	0.42	0.15, 0.63	0.0024	**	51
Age at sampling (y)	AXIN1	-0.41	-0.62, -0.15	0.0027	**	51
Age at sampling (y)	OSM	-0.41	-0.62, -0.14	0.0030	**	51
Age at sampling (y)	ST1A1	-0.40	-0.61, -0.12	0.0043	**	51
Age at sampling (y)	CCL25	0.36	0.09, 0.59	0.0088	**	51
Age at sampling (y)	CASP-8	-0.34	-0.57, -0.06	0.0143	*	51
Age at sampling (y)	CXCL6	-0.32	-0.56, -0.04	0.0203	*	51
Age at sampling (y)	Flt3L	0.32	0.04, 0.55	0.0216	*	51
Age at sampling (y)	IL-10RB	0.32	0.04, 0.55	0.0231	*	51
Age at sampling (y)	TGF-α	-0.32	-0.55, -0.04	0.0237	*	51
Age at sampling (y)	PD-L1	0.30	0.02, 0.54	0.0312	*	51
Age at sampling (y)	FGF-21	0.30	0.02, 0.54	0.0325	*	51
Age at sampling (y)	STAMBP	-0.29	-0.53, 0.00	0.0418	*	51
Age at sampling (y)	IFN-γ	0.28	0.00, 0.52	0.0457	*	51
Age at sampling (y)	CXCL10	0.28	-0.01, 0.52	0.0495	*	51
Age at diagnosis (y)	CXCL9	0.56	0.33, 0.73	<0.0001	****	51
Age at diagnosis (y)	OPG	0.56	0.33, 0.73	<0.0001	****	51
Age at diagnosis (y)	CSF-1	0.49	0.24, 0.68	0.0003	***	51
Age at diagnosis (y)	CDCP1	0.48	0.23, 0.67	0.0004	***	51
Age at diagnosis (y)	FGF-21	0.37	0.10, 0.59	0.0069	**	51
Age at diagnosis (y)	IL-10RB	0.36	0.09, 0.58	0.0092	**	51
Age at diagnosis (y)	EN-RAGE	-0.36	-0.58, -0.08	0.0097	**	51
Age at diagnosis (y)	FGF-23	0.35	0.08, 0.58	0.0113	*	51
Age at diagnosis (y)	ST1A1	-0.35	-0.57, -0.07	0.0131	*	51
Age at diagnosis (y)	SIRT2	-0.34	-0.57, -0.07	0.0140	*	51
Age at diagnosis (y)	AXIN1	-0.34	-0.57, -0.06	0.0142	*	51
Age at diagnosis (y)	CXCL10	0.32	0.04, 0.55	0.0218	*	51
Age at diagnosis (y)	IL-20	-0.32	-0.55, -0.04	0.0238	*	51
Age at diagnosis (y)	CCL25	0.31	0.03, 0.55	0.0251	*	51
Age at diagnosis (y)	CXCL6	-0.31	-0.55, -0.03	0.0261	*	51
Age at diagnosis (y)	TNFSF14	-0.31	-0.55, -0.03	0.0262	*	51
Age at diagnosis (y)	PD-L1	0.30	0.01, 0.53	0.0349	*	51
Age at diagnosis (y)	TWEAK	-0.29	-0.53, 0.00	0.0413	*	51
Age at diagnosis (y)	IL-15RA	0.28	0.00, 0.52	0.0435	*	51
Disease duration (m)	CCL23	0.46	0.20, 0.66	0.0007	***	51
Disease duration (m)	IL5	0.42	0.15, 0.63	0.0024	**	51
Disease duration (m)	CCL19	0.40	0.10, 0.60	0.0066	**	51
Disease duration (m)	TNFRSF9	0.40	0.10, 0.59	0.0077	**	51

Disease duration (m)	CDCP1	0.34	0.07, 0.57	0.0134	*	51
Disease duration (m)	CCL20	0.34	0.07, 0.57	0.0139	*	51
Disease duration (m)	CXCL9	0.34	0.07, 0.57	0.0140	*	51
Disease duration (m)	TSLP	0.33	0.05, 0.56	0.0171	*	51
Disease duration (m)	IL-12B	0.32	0.04, 0.55	0.0231	*	51
Disease duration (m)	CXCL10	0.31	0.03, 0.55	0.0266	*	51
Disease duration (m)	CCL28	0.31	0.03, 0.55	0.0267	*	51
Disease duration (m)	MCP-2	0.31	0.03, 0.54	0.0277	*	51
Disease duration (m)	CCL25	0.29	0.00, 0.53	0.0411	*	51
Disease duration (m)	LIF-R	0.29	0.00, 0.53	0.0427	*	51
Disease duration (m)	IL-10RB	0.28	0.00, 0.52	0.0446	*	51
Disease duration (m)	TNF-β	0.28	-0.01, 0.52	0.0495	*	51

Related to figure S2	Protein	Spearman r	95% CI	P value	Spearman Sign	# XY Pairs
Age at 1st manifestation (y)	CXCL9	0.52	0.28, 0.70	0.0001	***	50
Age at 1st manifestation (y)	OPG	0.50	0.25, 0.69	0.0002	***	50
Age at 1st manifestation (y)	CSF-1	0.48	0.22, 0.67	0.0005	***	50
Age at 1st manifestation (y)	CDCP1	0.45	0.18, 0.65	0.0012	**	50
Age at 1st manifestation (y)	EN-RAGE	-0.40	-0.60, -0.10	0.0068	**	50
Age at 1st manifestation (y)	AXIN1	-0.36	-0.59, -0.09	0.0094	**	50
Age at 1st manifestation (y)	IL-10RB	0.34	0.06, 0.57	0.0145	*	50
Age at 1st manifestation (y)	SIRT2	-0.34	-0.57, -0.06	0.0155	*	50
Age at 1st manifestation (y)	FGF-23	0.33	0.05, 0.56	0.0186	*	50
Age at 1st manifestation (y)	ST1A1	-0.33	-0.56, -0.05	0.0195	*	50
Age at 1st manifestation (y)	FGF-21	0.32	0.04, 0.56	0.0221	*	50
Age at 1st manifestation (y)	CXCL6	-0.32	-0.56, -0.04	0.0224	*	50
Age at 1st manifestation (y)	TNFSF14	-0.32	-0.55, -0.04	0.0238	*	50
Age at 1st manifestation (y)	CXCL10	0.31	0.03, 0.55	0.0270	*	50
Age at 1st manifestation (y)	CASP-8	-0.30	-0.54, -0.02	0.0339	*	50
Age at 1st manifestation (y)	PD-L1	0.29	0.01, 0.54	0.0379	*	50
Age at 1st manifestation (y)	IFN-γ	0.28	0.00, 0.53	0.0462	*	50
Age at 1st manifestation (y)	IL-20	-0.28	-0.52, 0.01	0.0491	*	50
Age at analysis (y)	CXCL9	0.56	0.33, 0.73	<0.0001	****	51
Age at analysis (y)	OPG	0.52	0.27, 0.70	0.0001	***	51
Age at analysis (y)	CDCP1	0.46	0.20, 0.65	0.0008	***	51
Age at analysis (y)	SIRT2	-0.44	-0.64, -0.18	0.0012	**	51
Age at analysis (y)	EN-RAGE	-0.43	-0.64, -0.17	0.0017	**	51
Age at analysis (y)	TNFSF14	-0.40	-0.61, -0.13	0.0040	**	51
Age at analysis (y)	AXIN1	-0.40	-0.61, -0.13	0.0040	**	51
Age at analysis (y)	OSM	-0.40	-0.60, -0.12	0.0050	**	51
Age at analysis (y)	FGF-23	0.37	0.10, 0.59	0.0069	**	51
Age at analysis (y)	ST1A1	-0.36	-0.59, -0.09	0.0086	**	51
Age at analysis (y)	CASP-8	-0.36	-0.58, -0.08	0.0099	**	51
Age at analysis (y)	CXCL6	-0.34	-0.57, -0.07	0.0132	*	51
Age at analysis (y)	CSF-1	0.34	0.06, 0.57	0.0144	*	51
Age at analysis (y)	STAMBP	-0.32	-0.56, -0.04	0.0207	*	51
Age at analysis (y)	Flt3L	0.30	0.02, 0.54	0.0321	*	51
Age at analysis (y)	LIF	-0.30	-0.54, -0.02	0.0337	*	51
Age at analysis (y)	CCL25	0.29	0.01, 0.53	0.0374	*	51

Age at analysis (y)	TGF-α	-0.29	-0.53, 0.00	0.0411	*	51

y: years; m: months; proteins are arranged as per decreasing strength of spearman R.

Related to figure 3	Protein	Spearman r	95% CI	P value	Spearman Sign	# XY Pairs
mRC	CXCL9	-0.44	-0.69, -0.09	0.0127	*	32
mRC	IFN-γ	0.41	0.07, 0.67	0.0182	*	32
mRC	CCL20	-0.41	-0.67, -0.06	0.0195	*	32
mRC	CDCP1	-0.41	-0.67, -0.06	0.0208	*	32
mRC	IL-17A	-0.40	-0.66, -0.04	0.0256	*	32
mRC	TNF	-0.37	-0.64, -0.01	0.0377	*	32
mRC	OPG	-0.35	-0.63, 0.00	0.0467	*	32
Totla INCAT at sampling	CXCL9	0.40	0.14, 0.62	0.0033	**	51
Totla INCAT at sampling	CCL11	0.40	0.10, 0.60	0.0065	**	51
Totla INCAT at sampling	TNFRSF9	0.32	0.04, 0.55	0.0232	*	51
Totla INCAT at sampling	OPG	0.31	0.03, 0.55	0.0262	*	51
Totla INCAT at sampling	CCL25	0.31	0.03, 0.55	0.0271	*	51
Totla INCAT at sampling	CDCP1	0.30	0.01, 0.54	0.0345	*	51
Totla INCAT at sampling	IL6	0.29	0.01, 0.53	0.0385	*	51
Totla INCAT at sampling	CCL20	0.28	0.00, 0.52	0.0472	*	51
Totla INCAT at sampling	IL-12B	0.28	-0.01, 0.52	0.0484	*	51

Related to figure 4	Protein	Spearman r	95% CI	P value	Spearman Sign	# XY Pairs
Calprotectin	TGF-α	0.73	0.50, 0.87	<0.0001	****	30
Calprotectin	TNFSF14	0.68	0.42, 0.84	<0.0001	****	30
Calprotectin	EN-RAGE	0.65	0.37, 0.82	<0.0001	****	30
Calprotectin	AXIN1	0.59	0.28, 0.79	0.0006	***	30
Calprotectin	SIRT2	0.56	0.24, 0.77	0.0014	**	30
Calprotectin	OSM	0.55	0.23, 0.76	0.0017	**	30
Calprotectin	HGF	0.52	0.19, 0.75	0.0030	**	30
Calprotectin	CASP-8	0.52	0.19, 0.75	0.0033	**	30
Calprotectin	ST1A1	0.52	0.18, 0.74	0.0035	**	30
Calprotectin	STAMBP	0.52	0.18, 0.74	0.0036	**	30
Calprotectin	CXCL1	0.51	0.18, 0.74	0.0037	**	30
Calprotectin	4E-BP1	0.50	0.16, 0.73	0.0048	**	30
Calprotectin	LIF	0.45	0.10, 0.71	0.0117	*	30
Calprotectin	VEGFA	0.45	0.10, 0.70	0.0125	*	30
Calprotectin	IL-15RA	0.45	0.10, 0.70	0.0127	*	30
Calprotectin	IL-20	0.44	0.09, 0.70	0.0142	*	30
Calprotectin	IL-18R1	0.43	0.07, 0.69	0.0172	*	30
Calprotectin	CCL20	0.40	0.03, 0.67	0.0300	*	30
Calprotectin	ARTN	0.39	0.03, 0.67	0.0316	*	30
Calprotectin	GDNF	0.39	0.03, 0.67	0.0321	*	30
Calprotectin	CD40	0.37	-0.01, 0.65	0.0470	*	30
NFL	OPG	0.58	0.31, 0.77	0.0002	***	37
NFL	NRTN	0.49	0.19, 0.71	0.0020	**	37
NFL	FGF-23	0.48	0.17, 0.70	0.0028	**	37
NFL	DNER	-0.46	-0.69, -0.15	0.0039	**	37

NFL	CXCL9	0.45	0.14, 0.68	0.0047	**	37
NFL	FGF-21	0.45	0.14, 0.68	0.0053	**	37
NFL	CDCP1	0.42	0.10, 0.66	0.0098	**	37
NFL	TRANCE	-0.42	-0.66, -0.10	0.0102	*	37
NFL	IL6	0.40	0.07, 0.64	0.0164	*	37
NFL	CSF-1	0.40	0.06, 0.64	0.0177	*	37
NFL	CD6	-0.37	-0.63, -0.05	0.0229	*	37
NFL	FGF-19	0.37	0.05, 0.63	0.0230	*	37
NFL	IL-1α	-0.37	-0.62, -0.04	0.0248	*	37
NFL	CCL3	0.35	0.02, 0.62	0.0312	*	37
NFL	ARTN	0.33	0.00, 0.60	0.0446	*	37
NFL	PD-L1	0.33	0.00, 0.60	0.0459	*	37
NFL	CD244	-0.33	-0.60, 0.01	0.0474	*	37
NFL	GDNF	0.33	-0.01, 0.60	0.0476	*	37
CSF	MMP-1	-0.46	-0.70, -0.13	0.0062	**	31
CSF	TWEAK	0.43	0.09, 0.67	0.0120	*	31
CSF	OPG	-0.40	-0.66, -0.06	0.0195	*	31
CSF	CCL28	-0.40	-0.66, -0.06	0.0196	*	31
CSF	CDCP1	-0.40	-0.65, -0.05	0.0214	*	31
CSF	CXCL9	-0.38	-0.65, -0.04	0.0247	*	31
CSF	IL6	-0.38	-0.65, -0.04	0.0251	*	31
CSF	CSF-1	-0.38	-0.64, -0.04	0.0256	*	31
CSF	IL-10RA	-0.36	-0.63, -0.01	0.0385	*	31
CSF	FGF-21	-0.35	-0.62, 0.00	0.0442	*	31

Proteins are arranged as per decreasing strength of spearman R. Proteins marked in bold text indicate moderate to high degree of correlation (i.e. Spearman R ≥ 0.4)
