# Supplementary material for: Serum inflammatory proteomic signatures define chronic inflammatory demyelinating polyneuropathy and inform on disease activity
Source: eBioMedicine. 2026 Jun 25;129:106348. doi: 10.1016/j.ebiom.2026.106348 (PMC13325459; doi:10.1016/j.ebiom.2026.106348)
Supplement: Supplementary Table S3 [file mmc3.pdf]

Table S3: Subgroup analysis with logistic regression Model 0, unadjusted for age and sex.

Related to figure 6

		Sex_Outcome_female			
Protein	OR	95 % CI		p-value	C-stat
FGF-19	1.71	1.02	2.87	0.0417	0.678
MCP-2	2.57	1.02	6.49	0.0452	0.665

		Electroneurography_Outcome_axonal feature			
Protein	OR	95 % CI		p-value	C-stat
uPA	0.07	0.01	0.62	0.0163	0.734
CD244	0.14	0.03	0.74	0.0198	0.701
β-NGF	0.01	0.00	0.48	0.0225	0.744
ST1A1	0.55	0.33	0.94	0.0281	0.682
DNER	0.10	0.01	0.80	0.0305	0.699
IL5	1.56	1.04	2.33	0.0314	0.668
IL-20RA	0.09	0.01	0.94	0.0441	0.715

		CIDP course_Outcome_unstable			
Protein	OR	95 % CI		p-value	C-stat
CCL4	2.33	1.17	4.63	0.0156	0.757
IL8	1.35	1.04	1.76	0.0248	0.724
CCL3	1.75	1.07	2.87	0.0256	0.741
FGF-21	1.40	1.04	1.90	0.0288	0.671
IL-17A	2.48	1.05	5.87	0.0392	0.68

		CIDP form_Outcome_acute onset			
Protein	OR	95 % CI		p-value	C-stat
MMP-10	0.29	0.10	0.84	0.0234	0.69

		IVIG termnated_Outcome_yes			
Protein	OR	95 % CI		p-value	C-stat
CCL23	0.02	0.00	0.24	0.0024	0.861
TNFRSF9	0.09	0.02	0.43	0.0029	0.859
IL6	0.35	0.17	0.72	0.0045	0.825
IL-12B	0.21	0.06	0.67	0.0083	0.8
IL-15RA	0.10	0.02	0.59	0.0109	0.768
IL18	0.20	0.06	0.70	0.0116	0.777
CDCP1	0.23	0.07	0.73	0.0126	0.73
CD5	0.14	0.03	0.67	0.0135	0.764
CXCL11	0.34	0.14	0.82	0.0158	0.73
IL10	0.30	0.11	0.81	0.0182	0.816
CXCL6	0.34	0.13	0.85	0.0219	0.739
IL33	0.08	0.01	0.75	0.0270	0.77
X4E-BP1	0.55	0.32	0.94	0.0287	0.73
CD244	0.15	0.03	0.87	0.0350	0.72
CSF-1	0.06	0.00	0.88	0.0402	0.675
PD-L1	0.16	0.03	0.92	0.0403	0.702
CXCL10	0.40	0.16	0.99	0.0467	0.689
CD8A	0.53	0.28	0.99	0.0476	0.739
CXCL1	0.41	0.17	0.99	0.0486	0.661

			SCIG status_Outcome_yes			
Protein	OR		95 % CI		p-value	C-stat
CCL20		1.87	1.08	3.24	0.025891	0.693
CCL19		1.71	1.02	2.84	0.040232	0.671
IL-12B		2.21	1.02	4.79	0.04534	0.699
DNER		0.13	0.02	0.96	0.045998	0.674

			Trigger_Outcome_vaccination			
Protein	OR		95 % CI		p-value	C-stat
CCL19		0.39	0.18	0.87	0.0210	0.833
CCL23		0.07	0.01	0.91	0.0424	0.782

Related to figure 7

			Electromyography_Outcome_axonal feature			
Variable	OR		95 % CI		p-value	C-stat
INCAT at follow-up		1.56	1.11	2.19	0.0103	0.747
mRC		0.92	0.86	0.99	0.0268	0.825
INCAT at sampling		1.55	1.04	2.33	0.0319	0.691
Age		1.05	1.00	1.10	0.0475	0.709

			CIDP course_Outcome_unstable			
Variable	OR		95 % CI		p-value	C-stat
INCAT at follow-up		1.51	1.09	2.10	0.0145	0.718

			CIDP form_Outcome_acute onset			
Variable	OR		95 % CI		p-value	C-stat
INCAT at sampling		1.57	1.01	2.43	0.0444	0.656

			IVIG termnated_Outcome_yes			
Variable	OR		95 % CI		p-value	C-stat
Disease duration (m)		0.97	0.94	0.99	0.0165	0.83

			SCIG status_Outcome_yes			
Variable	OR		95 % CI		p-value	C-stat
Disease duration (m)		1.02	1.01	1.04	0.009215	0.8

			Trigger_Outcome_vaccination			
Variable	OR		95 % CI		p-value	C-stat
Time from onset to diagnosis (m)		0.94	0.88	1.00	0.0463	0.824
